# Associations Between Steep Delay Discounting and Punishment Learning

**DOI:** 10.5334/cpsy.172

**Published:** 2026-09-01

**Authors:** Jeremy Haynes, Peter Kvam, Thomas Olino

**Affiliations:** 1 Georgia Southern University, US; 2 Ohio State University, US; 3 Temple University, US

**Keywords:** Iowa Gambling Task, Reward & Punishment Learning, Delay Discounting, Computational Modeling, Hierarchical Bayesian Analysis

## Abstract

Reward valuation and reward and punishment learning are important constructs for understanding and intervening on mental illness. These constructs are frequently measured with behavioral tasks such as intertemporal choice tasks, measuring delay discounting, and the Iowa Gambling Task (IGT), measuring reward and punishment learning. Delay discounting refers to how people value delayed outcomes, with steep discounting (i.e., delayed outcomes holding little value) considered a form of impulsive decision-making. Reward and punishment learning refers to how people adjust their decision behavior in response to rewards and punishments, with deficits indicating atypical reward and punishment processing related to clinical outcomes such as substance abuse. In this study, we tested the association between delay discounting and computational measures of reward and punishment learning in adults (*N* = 299) that completed an intertemporal choice task and the play-or-pass IGT. We fit a joint model to the task data to simultaneously estimate parameters from the hyperbolic discounting model (for the intertemporal choice task) and a reinforcement learning model (for the play-or-pass IGT). Our results indicate that steep delay discounting is associated with lower performance on the IGT, specifically through lower punishment learning. That is, people who do not value delayed outcomes also show less learning from punishment, resulting in worse performance in the IGT. Implications of these findings are discussed in the context of improving assessment of reward valuation and reward and punishment learning as constructs relevant to mental illness.

The National Institutes of Mental Health initiated the Research Domain Criteria (RDoC) as a framework for guiding research on the major psychological and biological systems involved in normal and abnormal human functioning (Insel, 2009). An important goal of introducing the RDoC was to understand the degree to which these underlying processes overlap and diverge from one another to improve our ability to identify patterns of dysfunction in mental illness (Cuthbert & Insel, 2013; Morris et al., 2022). In the present study, our goal was to explore how delay discounting, an RDoC measure of reward valuation, may be involved in decision-making on the Iowa Gambling Task (IGT), an RDoC measure of reward and punishment learning. Despite some inconsistencies, prior research suggests that delay discounting may be involved in learning on the IGT. However, decision-making on the IGT relies on multiple behavioral processes which are not captured by standard scoring procedures (Ahn et al., 2016). Therefore, it is unclear what specific aspects of learning on the IGT relate to delay discounting. To address this issue, we assessed associations between delay discounting and computationally derived measures of reward and punishment learning on the play-or-pass IGT. Computational methods allow us to break down IGT performance into more fine-grained patterns of behavior, and we expected that these more fine-grained measures would be differentially associated with delay discounting. Because measures of delay discounting and IGT performance are widely associated with psychopathology, identifying how delay discounting relates to specific aspects of learning on the IGT may have implications for understanding unique patterns of suboptimal behavior related to mental illness.

Delay discounting is an RDoC measure of reward valuation that characterizes the tendency to value delayed outcomes less than more immediate outcomes (Lempert et al., 2019; Odum, 2011). For example, $100 in 1 year is less valuable than $100 now. To accurately measure delay discounting, intertemporal choice tasks are used to assess preferences between smaller-sooner and larger-later rewards that vary in amounts and delays (e.g., $100 now or $200 in 1 year; Du et al., 2002). A pattern of preference for smaller-sooner over larger-later rewards indicates that delayed outcomes hold little value, referred to as steep delay discounting. In contrast, a pattern of preference for larger-later over smaller-sooner rewards indicates that delayed outcomes retain their value, referred to as shallow discounting.

Steep delay discounting is a form of impulsive decision-making widely associated with psychopathology. Meta-analyses show that steep discounting is related to attention-deficit/hyperactivity disorder (Jackson & MacKillop, 2016), post-traumatic stress disorder (Bird et al., 2024), multiple substance use disorders (MacKillop et al., 2011), among others (Amlung et al., 2019). These widespread associations have led some researchers to describe steep discounting as a trans-disease process (Bickel et al., 2012a, 2019); however, the specific role of steep discounting in these disorders is not entirely clear (Bailey et al., 2021). For addiction (e.g., substance use disorders), steep discounting is frequently described as a predisposition to preferring immediate, harmful reinforcers (e.g., pharmacological effects of a drug) while neglecting the delayed consequences of those reinforcers (e.g., unstable employment; Bickel et al., 2012a; Gray & MacKillop, 2015). Preclinical research (e.g., with non-human animals) and longitudinal studies (e.g., with developmental samples) are consistent with this perspective (Anker et al., 2009; Audrain-McGovern et al., 2009; Diergaarde et al., 2008; Perry et al., 2005); however, others have suggested that steep discounting may also be a consequence of excessive drug use (Volkow & Baler, 2015). For other psychiatric disorders, such as depression and anxiety, some have suggested steep discounting reflects a predisposition to neglecting prosocial reinforcers accumulated over time, contributing to symptoms of the disorder (Martínez-Loredo, 2023); however, we are unaware of research directly supporting this possibility. Instead, steep discounting is frequently described as a “marker” indicating dysfunctional reward valuation for internalizing forms of psychopathology like depression and anxiety (Lempert et al., 2019). In sum, steep discounting is widely considered an important risk factor for psychopathology; however, the role of steep discounting in these disorders remains unclear.

One approach to clarifying how delay discounting may be involved in psychopathology involves using multitask assessments to clarify how discounting relates to other presumably overlapping psychological constructs (Bailey et al., 2021). Here, we focus on how delay discounting may be related to learning from consequences on the Iowa Gambling Task. The Iowa Gambling Task (IGT; Bechara et al., 1994) is frequently used to measure reward and punishment learning by arranging a series of trials (usually 100–120) consisting of a choice between four decks of cards, with choices on each deck resulting in a gain (e.g., $100) and an occasional loss (e.g., –$150). Decks vary in the frequency and magnitude of gains and losses such that two decks are advantageous (i.e., “good”) and two decks are disadvantageous (i.e., “bad”). The good decks are characterized by small trial-level gains that accumulate over time which outweigh the occasional losses, and result in a net gain if played consistently. In contrast, the bad decks are characterized by large trial-level gains which are offset by larger and/or more frequent losses, and result in a net loss if played consistently. Participants are not told the outcomes beforehand; therefore, they must play on the decks to learn which are good and which are bad. Because the decks vary in their short- and long-term gains and losses, performance on the IGT has been described as a measure of how sensitive people are to consequences that accumulate over time (Bechara et al., 1994; Chiu et al., 2008). Assuming that better IGT performance relies on sensitivity to delayed outcomes, we would expect steep discounting (i.e., preference for immediacy) to be associated with failing to learn which decks are good and bad on the IGT.

Studies administering an intertemporal choice task and the IGT within the same participants suggest that steep discounting is associated with poor IGT performance. For example, Monterosso et al. (2001), in cocaine-dependent adults, and Olson et al. (2007), in adolescents, found that steep discounting was correlated with poor IGT performance. He et al. (2016) found that transcranial stimulation of the left dorsolateral prefrontal cortex (DLPFC) was associated with both increases in IGT performance and decreases in delay discounting. They speculated that stimulating the left DLPFC may have temporarily increased either impulse control or time perception, potentially increasing the temporal window through which individuals weigh delayed consequences on both tasks. Another study by Wölfling et al. (2020) found that people with gambling disorder show both steeper discounting and worse IGT performance relative to “healthy” controls. Aside from the broad construct of “impulsivity”, Wölfling et al. did not speculate what common mechanism may link steep discounting and worse IGT performance. Combined, these studies suggest that learning on the IGT is related to sensitivity to delayed consequences, as measured by delay discounting; however, some studies show little-to-no relation between the degree of discounting and IGT performance (Dom et al., 2007; Lamm et al., 2006), suggesting further research may be necessary to clarify these associations.

Besides studies examining delay discounting and IGT performance within the same participants, multiple lines of research suggest convergence between learning on the IGT and delay discounting. First, poor performance on the IGT is associated with similar forms of psychopathology as delay discounting, including for example depression (Siqueira et al., 2018; Wang et al., 2024) and substance use (Barry & Petry, 2008; Petry et al., 1998; Verdejo-Garcia et al., 2007; see Bickel et al., 2019 for a review on delay discounting in psychopathology; see Mukherjee & Kable, 2014 for a review & meta-analysis on IGT performance in psychopathology). Second, brain mechanisms involved in delay discounting overlap with those involved in the IGT, particularly the ventromedial prefrontal cortex (vmPFC). The vmPFC is thought to encode the subjective value of rewards (Hare et al., 2009; Kable & Glimcher, 2007), and dysfunction in the vmPFC is associated with both steeper discounting (Ciaramelli et al., 2021; Mok et al., 2021) and worse performance on the IGT (Bechara et al., 1994; Fellows & Farah, 2005; Mukherjee & Kable, 2014). Thus, converging behavioral, clinical, and neurobiological evidence suggests that delay discounting may be implicated in learning on the IGT, although some inconsistencies remain with respect to the behavioral evidence linking these two constructs (Dom et al., 2007; Lamm et al., 2006).

The research described above suggests that delay discounting, as a measure of reward valuation, is associated with reward and punishment learning on the Iowa Gambling Task (IGT); however, the specific learning processes on the IGT that contribute to this association are unclear. For example, performance on the IGT is typically aggregated into summary measures of behavior (e.g., net score across the whole session) describing how well participants did on the task, but the IGT is a complex task thought to rely on multiple behavioral processes that are not captured by these summary measures (Ahn et al., 2016; Mukherjee & Kable, 2014). For example, each deck within the IGT consists of both gains and losses which vary in magnitude and frequency; however, gains and losses influence behavior asymmetrically (Fontes & Shahan, 2021; Kahneman & Tversky, 1979) and sensitivity to win/loss frequency, irrespective of the magnitude of those wins and losses, has a strong effect on decision-making in the IGT (Chiu et al., 2008; Chiu & Lin, 2007; Steingroever et al., 2013; Wetzels et al., 2010). Failing to account for the asymmetry between learning from gains and losses and their frequencies could explain why some prior research has found no association between learning on the IGT and delay discounting (Dom et al., 2007; Lamm et al., 2006). For example, if the association between discounting and IGT performance is largely explained by differences in win/loss frequency sensitivity, we might expect weak associations when using standard IGT scoring procedures because the frequencies of wins and losses on the task are largely balanced across the good and bad decks.

In the present study, we sought to clarify the specific patterns of behavior on the Iowa Gambling Task (IGT) that would explain how learning on the IGT is related to sensitivity to immediate and delayed consequences, as measured by delay discounting. To do this, we used an existing dataset to assess the association between the degree of discounting from an intertemporal choice task and computationally derived measures of learning on the play-or-pass IGT. The play-or-pass IGT (Peters & Slovic, 2000) is a variant of the original task that involves presenting cards one at a time, allowing participants to either play and receive the card outcome, or pass and move on to the next trial. Although not central to the primary goals of this study — that is, assessing associations between discounting and computational measures of learning — this arrangement allows for summary measures of performance (e.g., good/bad play proportions) to distinguish whether shifts in playing on the good vs. bad decks reflect primarily reward learning (i.e., approaching the good decks) or punishment learning (i.e., avoiding the bad decks).^1^ Thus, in addition to assessing the association between computational measures of learning on the IGT and delay discounting, we also assessed whether associations between these computational measures and discounting could account for more molar (i.e., session-wide) patterns of behavior on the good versus the bad decks in the IGT.

To characterize learning on the play-or-pass Iowa Gambling Task (IGT), we used the Outcome Representation Learning (ORL) model. The ORL is a computational model developed for the original IGT (Haines et al., 2018), later adapted to the play-or-pass IGT (Haynes et al., 2024). The model characterizes task performance via four parameters: a reward learning rate, describing updating in response to gains; a punishment learning rate, describing updating in response to losses; a win/loss frequency sensitivity parameter, describing bias towards decks resulting in more frequent gains and/or away from decks with more frequent losses, regardless of magnitude; and a response bias parameter, describing a general tendency towards playing or passing, regardless of the outcomes associated with the decks. Broadly speaking, we hypothesized that people who showed steep discounting would perform worse on the task; however, computationally, we considered the possibility that steep discounting would be specifically associated with elevated reward and punishment learning rates. In particular, if we assume steep discounting indexes sensitivity to immediate consequences that would be reflected at the trial-level on the IGT, we would expect steep discounting to be associated with elevated reward and punishment learning rates as these parameters characterize how much people weight trial-level consequences in updating the value of a chosen option. However, an elevated punishment learning rate is associated with better performance on the play-or-pass IGT (see supplement for simulation study illustrating this pattern). Therefore, if participants who show steep discounting perform worse on the task, as expected, this could be due to an insensitivity to trial-level losses resulting in higher losses over time. Ultimately, our goal was to employ the ORL to identify specific patterns of behavior on the IGT, characterizing learning on the task, that may be differentially associated with steep delay discounting.

To more efficiently evaluate associations between ORL parameters and delay discounting, we estimated parameters using a joint modeling framework (Turner et al., 2013). Joint modeling involves estimating parameters from two or more models simultaneously by specifying a joint probabilistic structure in which data that inform parameter estimation in one model also inform parameter estimation in the other model (Haines et al., 2025; Zorowitz & Niv, 2023). Joint modeling is becoming an increasingly popular method of estimating computational parameters with higher reliability (Brown et al., 2020; Haynes et al., 2024; Karvelis et al., 2023; Sullivan-Toole et al., 2022), and has also been used to evaluate associations between psychological constructs. For example, Kvam et al. (2021) used joint modeling to assess whether decision-making on an intertemporal choice task and on the Cambridge Gambling Task (Rogers et al., 1999) represented overlapping forms of impulsivity. Participants completed both tasks and Kvam et al. estimated parameters from the hyperbolic discounting model (Mazur, 1987) simultaneously with parameters from the Luce choice/bet utility model (Romeu et al., 2020). Their findings suggested that the intertemporal choice task and the Cambridge Gambling Task measure two unique forms of impulsivity, impulsive choice and impulsive action, which had unique associations with substance abuse (e.g., heroin vs. amphetamine use). Thus, these studies show the utility of joint modeling for not only assessing test-retest reliability, but also for assessing convergence (or divergence) across psychological constructs (e.g., impulsive choice & impulsive action).

In sum, our goal was to clarify the role of delay discounting in learning on the play-or-pass Iowa Gambling Task using state-of-the-art computational methods. As noted, we used an existing dataset in which participants completed an intertemporal choice task and the play-or-pass IGT. Data from the play-or-pass IGT have been previously published (Haynes et al., 2025). For transparency, it should be noted that the previous study examined history of psychopathology on IGT performance and found that people with a current and past history of anxiety (combined) tend to show elevated punishment learning on the task (see Haynes et al., 2025 for details). The focus of this study was to clarify if and how delay discounting may be implicated in computational measures of learning on the play-or-pass IGT. Thus, to avoid redundancy in publications, we focus our analyses on the behavioral associations between delay discounting and IGT performance only. Given that steep discounting and poor IGT performance are both associated with socially relevant individual differences (e.g., depression & substance use disorder; Bickel et al., 2019; Mukherjee & Kable, 2014), we sought to provide a preliminary analysis of how delay discounting, as a measure of reward valuation, could inform our understanding of reward and punishment learning as constructs involved in mental illness, defined by the RDoC (Insel, 2009).

## Method

We used data from a three-year study examining various facets of the Positive and Negative Valence Systems in youth and their parents. For the purposes of examining the relation between delay discounting and reward and punishment learning, we focus on data collected from a standard intertemporal choice task and the play-or-pass Iowa Gambling Task (IGT) at baseline for parents. Data from the play-or-pass IGT have been presented elsewhere (Haynes et al., 2025). All procedures were approved by the Temple University institutional review board (IRB Protocol #23174 originally approved on 08/31/2015) and conducted in accordance with the Declaration of Helsinki. All participants provided informed consent, and their privacy has been upheld.

### Participants

Participants were adults with a child between 9–10 or 12–13 years, recruited from the community within a 30-mile radius of a metropolitan university in the northeastern United States. Only English-speaking families were included, and families with children prescribed psychotropic medications (except for ADHD medication), met diagnostic criteria for a serious neurological illness, or had learning or developmental disabilities at baseline were excluded. Children’s intellectual ability was tested at baseline using the Kaufman Brief Intelligence Test (KBIT-2; Naugle et al., 1993), and families with children whose KBIT-2 scores of overall intelligence levels fell two or more standard deviations below the mean were excluded (KBIT-2 FSIQ < 70). Finally, parents with a lifetime history of bipolar spectrum disorder or psychosis spectrum disorder were not eligible for participation and led to removing the family from the study. The final sample size was 299 parents, inclusive of mothers and fathers. Demographic characteristics of the sample are presented in Table 1.

**Table 1:** Demographic Characteristics of Sample.


	*N* = 299

*M* (*SD*) age in years	39.98 (7.11)

Male	78 (26%)

Female	221 (74%)

Race	

Asian	4 (1%)

Black or African American	94 (31%)

Biracial	9 (3%)

White	96 (32%)

Other	12 (4%)

Not disclosed	84 (28%)

Ethnicity	

Hispanic	21 (7%)

Non-Hispanic	269 (90%)

Not disclosed	9 (3%)


### Procedure

Participants completed a series of behavioral tasks, self-report questionnaires, and neurological measures. For brevity, we only describe the relevant measures for this study: performance on the play-or-pass Iowa Gambling Task and on the intertemporal choice task at baseline. Participants were compensated with $95 following the baseline session.

#### Play-or-Pass Iowa Gambling Task

The play-or-pass version of the Iowa Gambling Task (IGT) was administered using E-Prime Stimulus Presentation Software (Peters & Slovic, 2000; Schneider et al., 2002). All outcomes in the task were hypothetical. Participants began the task with a “bank” of $2000 and were presented with four decks of cards. On each trial, a single deck was highlighted, and participants had the opportunity to “play” or “pass” on the highlighted deck. If a participant played on the deck, they would receive either a monetary gain, loss, or a neutral outcome (i.e., $0 change) from the drawn card. If a participant passed on the deck, they moved onto the next trial. Participants completed 120 trials with each deck presented 30 times each. Each deck was associated with a different distribution of gains and losses such that the long-run average expected value was –$25 for Decks A and B (i.e., the disadvantageous/bad decks) and $20 and $25 for Decks C and D, respectively (i.e., the advantageous/good decks). We used two versions of the task that differed only in the order in which cards were presented. Each version was associated with a fixed sequence of cards, and participants were assigned to one of those versions using counterbalancing. Participants were not informed of the payout distribution nor the sequence of decks and cards that would be presented, requiring participants to sample the cards from each deck and learn the outcomes associated with each deck. Trials ended if participants did not respond within 4 seconds which were scored as passing on the card. Participants were told that their earnings in the task would be exchanged for a real cash bonus; however, all participants received $10 for completing the behavioral tasks.

#### Intertemporal Choice Task

The intertemporal choice task was administered using E-Prime. Participants were asked their preference between a smaller-immediate monetary reward (e.g., $500 now) and a larger-delayed monetary reward (e.g., $1000 in 1 year). As with the play-or-pass IGT, all outcomes were hypothetical. The task employed an adjusting-amount algorithm to obtain indifference points at five delays, presented in blocks of 9–11 trials each (Du et al., 2002). For each delay block, the starting value of the smaller-immediate reward was $200, $500, or $800, randomly determined, and the value of the larger-delayed reward was always $1000. Across trials within a delay block, the amount of the immediate reward was increased or decreased based on participants’ previous choices until converging on an indifference point (see supplement for details), defined as the subjective value of the larger, delayed reward. Delays were 1 day, 1 week, 1 month, 3 months, 6 months, and 1 year, presented randomly across delay blocks.

### Data Analysis

We conducted a preliminary analysis to determine whether participants were sensitive to the long-term outcomes associated with the decks on the play-or-pass Iowa Gambling Task (IGT). To do this, we tested whether play proportions varied across decks with a generalized linear mixed model (GLMM) fit using the lme4 package (Bates et al., 2015). We used a binomial link function to fit the GLMM to the number of plays for each deck (out of 30 total trials for each deck). We included a fixed effect of deck (categorical) and random intercepts for participants: *Plays* ~ *Deck* + (1|Participant). We evaluated the significance of the fixed effect of deck with a Wald test using the car package (Fox & Weisberg, 2019) with Bonferroni-corrected follow-up comparisons conducted using the emmeans package (Lenth & Piaskowski, 2026). Results of the GLMM are presented with posterior predictive checks (see below for details) to support the visual analysis of the data.

Next, we used a hierarchical Bayesian approach to fit a joint model composed of parameters from the hyperbolic discounting model and the play-or-pass Outcome Representation Learning (ORL) model. The joint model was fit in Stan (v. 2.36.0; Stan Development Team, 2023), a probabilistic programming language which estimates parameters using Hamiltonian Monte Carlo, a variant of Markov chain Monte Carlo (MCMC) to sample from high-dimensional probabilistic models. We used R (v. 4.4.3; R Core Team, 2025) to interface with Stan via the CmdStanR package (v. 0.9.0; Gabry et al., 2025). Person level parameters were drawn from group-level distributions to leverage hierarchical pooling such that the group-level parameters served as priors for the person level parameters. For concision and clarity, we present the centered parameterizations of the person level parameters below; however, the models were implemented using equivalent non-centered parameterizations to increase efficiency and model convergence (Stan Development Team, n.d.).

We sampled each model using 4 chains, each with 1000 warmup samples and 4000 target samples (5000 iterations/chain; cf. Sullivan-Toole et al., 2022). Convergence of target distributions were inspected visually with trace-plots and for each parameter numerically with $\hat{R}$ values (Gelman & Rubin, 1992). $\hat{R}$ values were all below 1.1, indicating that the variance between chains did not outweigh the variance within chains (i.e., convergence). We performed posterior predictive checks to assess how well model-predictions aligned with the observed data, discussed in further detail below. Finally, we performed parameter recovery diagnostics, displayed in detail in the supplement. In general, parameters showed adequate recovery; however, reward learning rates from the Outcome Representation Learning model showed the poorest recovery (*r* = .48). Implications of this suboptimal parameter recovery are discussed in the Discussion.

#### Hyperbolic Discounting Model

We estimated the degree of discounting by assuming hyperbolic decay of value as delay increases (Mazur, 1987):

Equation 1 $$V=\frac{A}{1+k\cdot D}$$

where *V* is the discounted value of the delayed outcome, *A* is the amount of the delayed outcome, *k* is a free parameter describing the degree of discounting, and *D* is the delay. To estimate *k* values, we used an approach described by Patt et al. (2021) in which, by normalizing indifference points on a 0 to 1 scale (hereafter referred to as ′V), the hyperbolic discounting model can be expressed as a logistic function:

Equation 2 $${\;}^{'}V=\frac{1}{1+\exp\left(\text{log}k+\text{log}D\right)}$$

By rearranging Equation 2 (see supplement), we can estimate *k* from the normalized indifference points using a linear model:

Equation 3 $$\text{log}\frac{1-{\;}^{'}V_{\mathrm{ij}}}{{\;}^{'}V_{\mathrm{ij}}}\sim \mathrm{Normal}(\text{log}k_{i}+\text{log}D_{j},\phi_{i})$$

where *k* is as described above and *ϕ* is an error term that describes variability in indifference points, with the subscripts *i* and *j* referring to individual participants and delays, respectively. Equation 3 was used to estimate person level *k*- and *ϕ*-values, drawn from separate lognormal group-level distributions (cf. Kvam et al., 2021; Vincent, 2016; Young, 2017):

$$\begin{array}{l} \text{log}k_{i}\sim \;\text{Normal}(\mu_{\mathrm{logk}},\sigma_{\mathrm{logk}})\\ \text{log}\phi_{i}\sim \;\text{Normal}(\mu_{\text{log}\phi },\sigma_{\text{log}\phi }) \end{array}$$

where the *µ*s and *σ*s are the group-level means and standard deviations, respectively. For the group-level parameters, we specified the following priors:

$$\begin{array}{c} \mu_{\text{log}k}\sim \text{Normal}\left(0,1\right)\\ \mu_{\text{log}\phi }\sim \text{Normal}\left(0,0.5\right)\\ \sigma_{\text{log}k},\sigma_{\text{log}\phi }\sim \text{Half-Normal}\left(0.5\right) \end{array}$$

#### Play-or-Pass ORL Model

For the play-or-pass Iowa Gambling Task (IGT), we estimated parameters from the play-or-pass Outcome Representation Learning (ORL) model using the trial-level choice data (Haines et al., 2018; Haynes et al., 2024). Specifically, we modeled choices to play according to a Bernoulli likelihood function:

Equation 4 $$Y_{i,j}(t)\sim \text{Bernoulli}\left(\frac{1}{1+\exp\left(-V_{i,j}\left(t\right)\right)}\right)$$

where *Y_i,j_*(*t*) refers playing (*Y* = 1) or passing (*Y* = 0) on the *j^th^* deck presented on trial *t* and *V_i,j_*(*t*) refers to the value of playing on deck *j* presented on trial *t*, with the subscript *i* indexing individual participants. The model updates value (*V*) from trial to trial as the following:

Equation 5 $$V_{i,j}\left(t+1\right)=EV_{i,j}\left(t+1\right)+EF_{i,j}\left(t+1\right)\cdot \beta f_{i}+\beta b_{i}$$

where *EV_i, j_* (*t* + 1) is the updated expected value of deck *j* after trial *t, EF_i, j_* (*t* + 1) is the updated expected win/loss frequency of deck *j* after trial *t, βf_i_* is a win/loss frequency sensitivity parameter, and *βb_i_* is a response bias parameter.

Expected value and expected win/loss frequency update according to reinforcement learning based algorithms. For expected value, we use the following algorithm:

Equation 6 $$EV_{i,j}\left(t+1\right)=\{\begin{array}{cc} EV_{i,j}\left(t\right)+A_{\text{rew},i}\cdot (x\left(t\right)-EV_{i,j}\left(t\right)) & \mathrm{if}Y_{i,j}\left(t\right)=1\text{ and }x\left(t\right)\geq 0\\ EV_{i,j}\left(t\right)+A_{\text{pun},i}\cdot (x\left(t\right)-EV_{i,j}\left(t\right)) & \mathrm{if}Y_{i,j}\left(t\right)=1\text{ and }x\left(t\right)<0\\ EV_{i,j}\left(t\right) & \mathrm{if}Y_{i,j}\left(t\right)=0 \end{array}$$

where *EV_i, j_* (*t*) is the expected value of deck *j* on trial *t, x*(*t*) is the outcome on trial *t* (e.g., win $200 = +200; lose $200 = –200), *A_rew,i_* is a learning rate for gains or neutral outcomes (i.e., *x*(*t*) ≥ 0), and *A_pun,i_* is a learning rate for losses (i.e., *x*(*t*) < 0). For expected win/loss frequency, we use a similar algorithm:

Equation 7 $$\begin{array}{l} EF_{i,j}\left(t+1\right)\\ =\{\begin{array}{cc} EF_{i,j}\left(t\right)+A_{\text{rew},i}\cdot (\text{sgn}\left(x\left(t\right)\right)-EF_{i,j}\left(t\right)) & \mathrm{if}Y_{i,j}\left(t\right)=1\;\text{ and }x\left(t\right)\geq 0\\ EF_{i,j}\left(t\right)+A_{\text{pun},i}\cdot (\text{sgn}\left(x\left(t\right)\right)-EF_{i,j}\left(t\right)) & \mathrm{if}Y_{i,j}\left(t\right)=1\text{ and }x\left(t\right)<0\\ EF_{i,j}\left(t\right) & \mathrm{if}Y_{i,j}\left(t\right)=0 \end{array} \end{array}$$

where *EF_i, j_* (*t*) is the expected win/loss frequency of deck *j* on trial *t, sgn*(*x*(*t*)) is the outcome on trial *t* transformed using the signum function (+1 for gains, 0 for neutral outcomes, & –1 for losses), and *A_rew,i_* and *A_pun,i_* are as described above. It is worth noting that *EV* and *EF* only update when participants play (i.e., *Y*(*t*) = 1); when participants pass (i.e., *Y*(*t*) = 0), no updating occurs. For unchosen decks, expected win/loss frequencies update according to a fictive learning rule, based on the original ORL model (Haines et al., 2018):

Equation 8 $$\begin{array}{l} EF_{i,{\;}^{'}j}\left(t+1\right)\\ =\{\begin{array}{cc} EF_{i,{\;}^{'}j}\left(t\right)+A_{\text{pun},i\;}\cdot \left(\frac{-\text{sgn}\left(x\left(t\right)\right)}{C}-EF_{i,{\;}^{'}j}\left(t\right)\right) & \mathrm{if}Y_{i,j}\left(t\right)=1\text{ and }x\left(t\right)\geq 0\\ EF_{i,{\;}^{'}j}\left(t\right)+A_{\text{rew},i\;}\cdot \left(\frac{-\text{sgn}\left(x\left(t\right)\right)}{C}-EF_{i,{\;}^{'}j}\left(t\right)\right) & \mathrm{if}Y_{i,j}\left(t\right)=1\text{ and }x\left(t\right)<0\\ EF_{i,{\;}^{'}j}\left(t\right) & \mathrm{if}Y_{i,j}\left(t\right)=0 \end{array} \end{array}$$

where $EF_{i{,}^{'}j}(t)$ is the expected win/loss frequency for unchosen decks on trial *t, C* is the number of other decks available (i.e., *C* = 3), and all other terms are as described above. In total, the ORL model includes four parameters: reward learning rate (*A_rew_*), punishment learning rate (*A_pun_*), win/loss frequency sensitivity (*βf*), and response bias (*βb*).

In the joint model, person-level ORL parameters were drawn from group-level distributions:

$$\begin{array}{c} \Phi^{-1}(A_{\text{rew},i}\;\text{/}\mathrm{scale})\sim \text{Normal}(\mu_{{\;}^{'}A_{\text{rew}},i},\sigma_{{\;}^{'}A_{\text{rew}}})\\ \Phi^{-1}(A_{\text{pen},i}\text{/}\mathrm{scale})\sim \text{Normal}(\mu_{{\;}^{'}A_{\text{pen},i}},\sigma_{{\;}^{'}A_{\text{pen}}})\\ \beta f_{i}\sim \text{Normal}(\mu_{\beta f,i},\sigma_{\beta f})\\ \beta b_{i}\sim \text{Normal}(\mu_{\beta b,i},\sigma_{\beta b}) \end{array}$$

where the *μ*s represent group-level models, described below, and the *σ*s represent group-level standard deviations. Note that because reward and punishment learning rates (*A_rew_* & *A_pun_*) are bounded between 0 and 1 in the ORL algorithm, person-level learning rates were drawn from normal distributions and subsequently transformed using the cumulative distribution function (*scale* = 1) for the standard normal distribution—i.e., *A_rew_* = Φ(`*A_rew_*) and *A_pun_* = Φ(`*A_pun_*).

We chose the ORL model as, to our knowledge, it is the only computational model available for the play-or-pass IGT. Per recommendations from two helpful reviewers, we examined how well the ORL fit the data compared to simpler reinforcement learning models: one model with a single learning rate for both gains and losses, and another model with separate learning rates for gains and losses. Details of the models and results are presented in the supplement. In brief, the ORL outperformed the simpler reinforcement learning models; therefore, we proceeded with using the ORL in the joint model.

#### Joint Model

The joint model consisted of constructing the group-level ORL parameters (i.e., the *μ*s) as a function of the discounting rates from the hyperbolic discounting model (i.e., log *k* values). To do this, we used an approach based on Kvam et al. (2021) which employed linear functions for the group-level ORL parameters:

Equation 9 $$\begin{array}{c} \mu_{{\;}^{'}A_{\text{rew}},i}=\gamma_{{\;}^{'}A_{\text{rew}}0}+\gamma_{{\;}^{'}A_{\text{rew}}1}\cdot \text{log}k_{i}\\ \mu_{{\;}^{'}A_{\text{pen}},i}=\gamma_{{\;}^{'}A_{\text{pen}}0}+\gamma_{{\;}^{'}A_{\text{pen}}1}\cdot \text{log}k_{i}\\ \mu_{\beta f,i}=\gamma_{\beta f0}+\gamma_{\beta f1}\cdot \text{log}k_{i}\\ \mu_{\beta b,i}=\gamma_{\beta b0}+\gamma_{\beta b1}\cdot \text{log}k_{i} \end{array}$$

where the *γ*_0_s represent the group-level means for the corresponding ORL parameter when the discounting parameter, log *k*, is 0 (i.e., an intercept) and the *γ*_1_s represent the group-level effects of log *k* on the corresponding ORL parameter (i.e., a slope effect). Log *k* values were used based on convention (Kvam et al., 2021; Vincent, 2016) because raw *k* values are right skewed (Baker et al., 2003; Kirby et al., 1999). Equation 9 represents the most important piece of the joint model as it allowed us to not only estimate parameters from the hyperbolic discounting model and the ORL model simultaneously but also estimate how parameters from each model were related to one another via the *γ*_1_ parameters (similar to the exploratory joint modeling approach implemented by Wall et al., 2021).

For the group-level parameters, we used the following priors:

$$\begin{array}{c} \gamma_{{\;}^{'}A_{\text{rew}}0},\gamma_{{\;}^{'}A_{\text{pen}}0},\;\gamma_{\beta f0},\;\gamma_{\beta b0}\sim \text{Normal}\left(0,1\right)\\ \gamma_{{\;}^{'}A_{\text{rew}}1},\;\gamma_{{\;}^{'}A_{\text{pen}}1},\;\gamma_{\beta f1},\;\gamma_{\beta b1}\sim \text{Normal}\left(0,0.5\right)\\ \sigma_{{\;}^{'}A_{\text{rew}}}\sigma_{{\;}^{'}A_{\text{pen}}}\sim \text{Half-Normal}\left(0.2\right)\\ \sigma_{\beta f},\sigma_{\beta b}\sim \text{Half-Cauchy}\left(1\right) \end{array}$$

#### Prior Choices

We chose priors based on literature and simulations. For the linear functions connecting parameters from the hyperbolic discounting model to the parameters from the ORL model, we chose weakly informative priors for the intercept coefficients (i.e., the *γ_0_*s) with more informative priors on the slope coefficients (i.e., the *γ_1_*s). More informative priors on the slope coefficients were chosen to improve model convergence. These stronger priors on the slope coefficients are also more conservative, such that the effects of interest (i.e., cross-task associations) are drawn closer to 0 (i.e., towards no association). For the ORL model parameters, we chose weakly informative priors based on previous research using this model (Haynes et al., 2024, 2025). Finally, we chose priors for parameters of the hyperbolic discounting model using simulations from the priors. We describe these simulations in detail in the supplement, but in brief, the simulations revealed that prior probabilities associated with person-level indifference points can vary substantially as a function of the group-level log *k* prior. To address concerns regarding whether the choice of one prior over another would meaningfully influence our results, we fit the joint model using different group-level log *k* priors and found the same pattern of findings described in the results with each set of priors. We ultimately present results from the *μ*_log_*_k_* ~ Normal(0,1) for simplicity, but it should be noted that this is not a weakly informative prior (see supplement for details).

#### Posterior Predictive Checks

After fitting the joint model, we assessed how well predictions from the model aligned with the observed data using posterior predictive checks (Wilson & Collins, 2019). To do this, we simulated data (IGT choices & indifference points) from each iteration of the posterior distribution for individual participants. For the play-or-pass ORL, we calculated group-level play proportions for each trial and deck by iteration. Next, we calculated a posterior “*p*-value” (Gelman et al., 1995) by computing the proportion of simulated play proportions falling below the observed group-level play proportion across trials and decks. Accordingly, a well-fitting model would have a *p*-value of .5 (i.e., producing predictions centered around the observed data) whereas a poor-fitting model would have a *p*-value closer to 0, indicating posterior predictions underestimate the data, or 1, indicating posterior predictions overestimate the data. We used a similar approach for the hyperbolic discounting model; we calculated group-level median indifference points for each delay by iteration. Next, we calculated a posterior *p*-value by computing the proportion of simulated median indifference points falling below the observed median indifference points across delays. Finally, we graphically checked how well simulated data aligned with the observed data by plotting the group-level model-predictions with the observed data.

#### Statistical Inference

Because the joint model was fit using a Bayesian approach, we based our inferences on evaluating the posterior distributions of the parameters and on Bayes factors. Specifically, distributions of *γ_1_*s in which the 95% highest density intervals (HDIs) did not overlap with 0 were considered evidence in favor of an association between log *k* values and the corresponding ORL parameter. In addition, we calculated approximate Bayes Factors using the Savage-Dickey method (Wagenmakers et al., 2010) to quantify evidence in favor of an association between log *k* values and the corresponding ORL parameters (i.e., *γ_1_* ≠ 0) versus no association between the parameters (i.e., *γ_1_* = 0; see Kvam et al., 2021 for a similar application of Bayes factors). Bayes factors of 1.0 indicate inconclusive evidence for an association between parameters, with BF_10_ values less than 1.0 indicating evidence of no association between parameters, values 3–10 indicating “moderate” evidence of an association, values 10–30 indicating “strong” evidence of an association, and values greater than 30 indicating “very strong” evidence in favor of an association (Jeffreys, 1961; Lee & Wagenmakers, 2014).

To further contextualize the between-task associations, we calculated follow-up correlations between discounting parameters and ORL parameters, and between discounting parameters and behavioral measures of IGT performance. First, we calculated correlations between log *k* values and ORL parameters as more intuitive measures of the associations between parameters. These correlations are presented purely for ease of interpretation, but they otherwise provide the same information as the *γ_1_* parameters. To do this, we calculated correlations iteration-by-iteration from the posterior distributions of the person-level parameters to generate posterior distributions of correlation coefficients. These distributions are summarized in Table 2 and displayed visually in the supplement. In addition, we computed correlations between posterior means of the person-level log *k* values and summary measures of IGT performance: net earnings on the task and session-wide play proportions on the good and bad decks. For bounded measures (e.g., play proportions), we used Spearman non-parametric correlations, indicated with the subscript S. Correlations were considered statistically significant at the α = .05 level.

**Table 2:** Descriptive Statistics of Posterior Distributions.


PARAMETER	*R*	*γ*_0_	*γ*_1_	*μ*

*A_rew_*	–.21 [–.51,.08]	–1.21 [–1.29,–1.14]	–0.02 [–0.06,0.01]	0.12 [0.10,0.13]

*A_pun_*	**–.40 [–.52,–.29]**	–1.42 [–1.48,–1.36]	**–0.07 [–0.10,–0.04]**	0.09 [0.08,0.10]

*βf*	**–.21 [–.30,–.12]**	3.42 [2.98,3.91]	**–0.32 [–0.54,–0.09]**	3.36 [2.89,3.83]

*βb*	**–.20 [–.30,–.08]**	1.05 [0.94,1.15]	**–0.07 [–0.12,–0.01]**	1.03 [0.92,1.14]

Log *k*				0.21 [–0.05,0.47]

Log				0.58 [0.53,0.64]


*Note*. Group-level posterior means ±95% HDI. *μ*s for the ORL parameters are posterior means at the group-level mean log *k*. *μ*s for *A_rew_* and *A_pun_* are presented on the transformed scale (i.e., bounded between 0 & 1). Bold *r*s and *γ*_1_s indicate correlations/effects in which the 95% HDIs did not overlap with 0, indicating evidence of an association between log *k* and the ORL parameter.

## Results

### Posterior Predictive Checks

**Figure 1:**
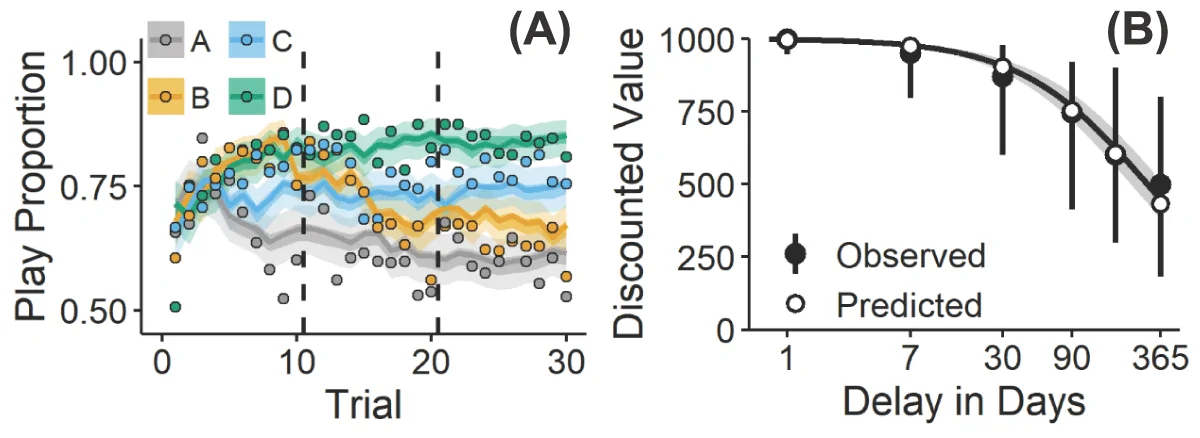
Observed & Model-Predicted Data. *Note*. **(A)** Group-level mean play proportions across trials for each deck in the play-or-pass Iowa Gambling Task. Datapoints represent observed play proportions and lines represent play-or-pass ORL model-predicted play proportions. Error bands represent 50% (darker) and 95% (lighter) HDIs. **(B)** Discounted value of the delayed hypothetical $1000 as a function of delay. Delays are displayed on a logarithmic scale to illustrate data at shorter delays. Observed data represent median indifference points with interquartile ranges. Predicted data represent posterior means of the model-predicted group-level medians. Curve represents discounted value according to the group-level mean *k* value ±95% HDI (error band).

In general, participants showed typical performance on the play-or-pass Iowa Gambling Task (IGT) and on the intertemporal choice (ITC) task, and the model captured these patterns well. To illustrate, Figure 1 shows observed- and model-predicted data for the play-or-pass IGT (Figure 1A) and the ITC task (Figure 1B). On the IGT, participants were sensitive to the contingencies associated with each deck, such that across trials, participants avoided the bad decks (i.e., passed on decks A & B) and approached the good decks (i.e., played on decks C & D). The GLMM on the number of plays across decks revealed a significant main effect of deck χ^2^(3) = 972.22, *p* < .001. Follow-up comparisons showed that participants played on the good decks (decks C & D) more often than on the bad decks (decks A & B), all *p*s < .001. Among the two good decks, participants chose deck D, with the most frequent wins, more often than deck C, *p* < .001. In addition, among the two bad decks, participants chose deck A, with the most frequent losses, less often than deck B, *p* < .001. For the ITC task, as the delay to the $1000 reward increased, the discounted value of that outcome (i.e., the indifference points) systematically decreased. As Figure 1 shows, the joint model captured these patterns of behavior well within each task. Likewise, the posterior *p*-values for the IGT and ITC task data were .498 and .465, respectively, indicating that posterior predictions aligned with the observed data.^2^

### Cross-Task Associations

**Figure 2:**
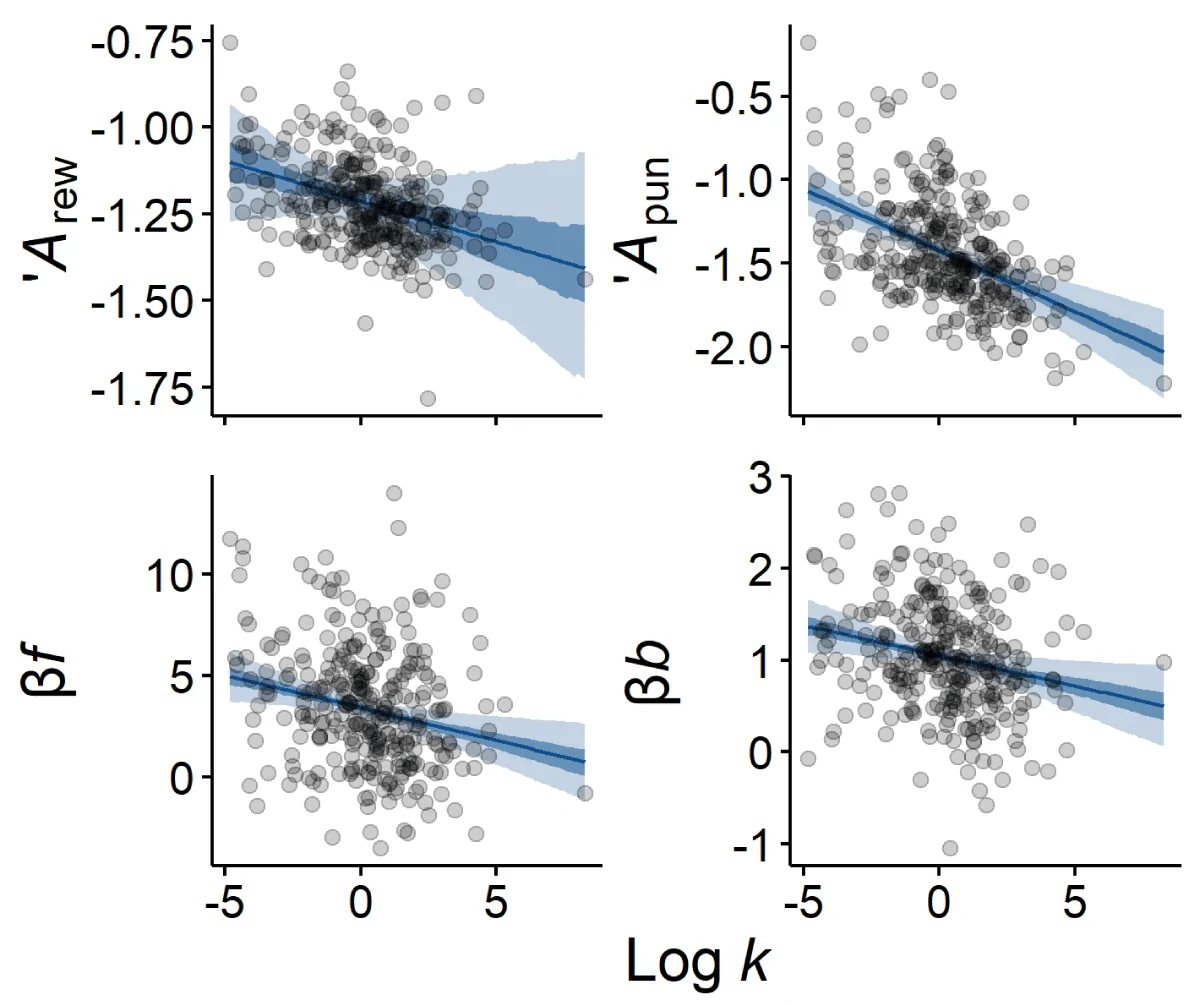
ORL Parameters as a Function of Log ks. *Note*. Parameters from the joint model with log *k* values on the *x*-axis and ORL parameters on the *y*-axis. Regression lines were constructed from the *γ*s, estimating the relation between log *k* and ORL parameters. Darker and lighter shaded areas represent 50% and 95% HDIs, respectively. Datapoints represent posterior means of individual participant parameters.

Overall, steep delay discounting (i.e., a higher log *k*) was associated with lower reward and punishment learning rates (*A_rew_* & *A_pun_*), win/loss frequency sensitivities (*βf*), and response biases (*βb*). To illustrate, Figure 2 displays point estimates of the ORL parameters as a function of the point estimates of the log *k* values for individual participants. Point estimates represent posterior means of the person-level parameters. Each panel shows a negative association between log *k* values and the corresponding ORL parameter, but further examination of the joint model parameters is necessary to identify evidence for or against these associations.

**Figure 3:**
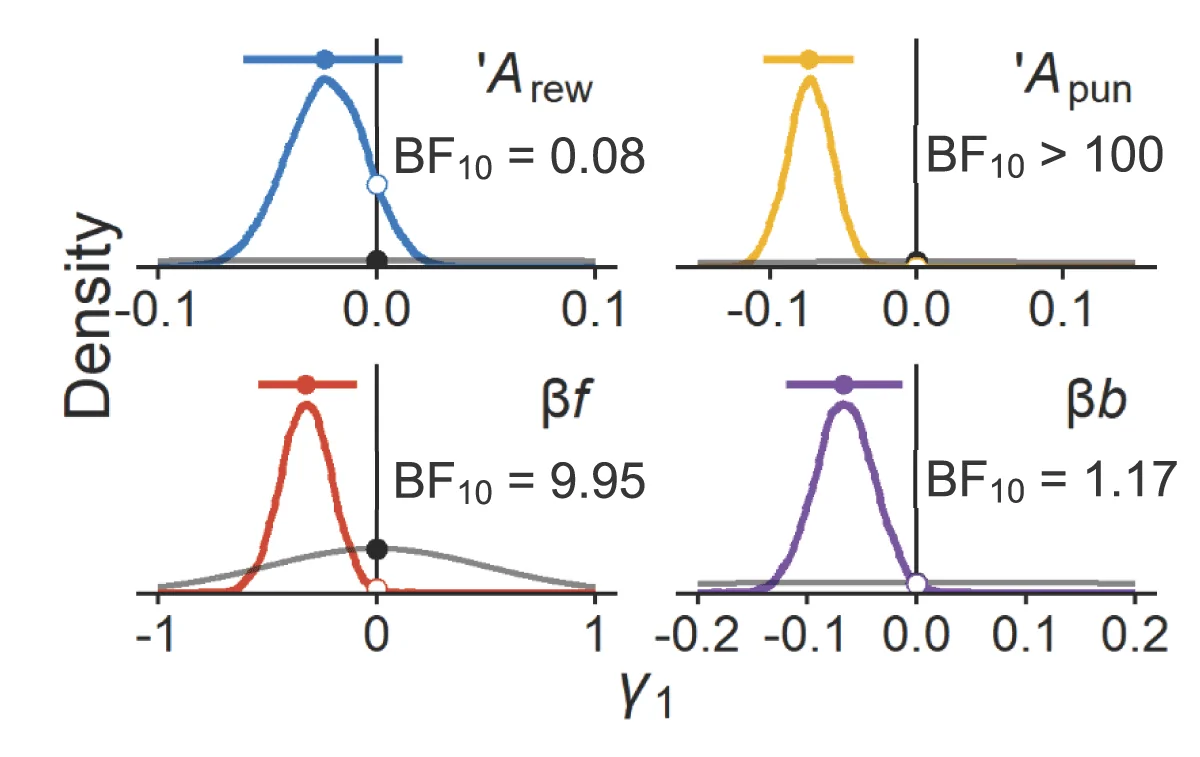
Cross-Task Associations Between ORL Parameters & Log ks. *Note*. Posterior densities of the *γ*_1_ parameters characterizing the association between log *k* values and ORL parameters. Posterior means (datapoints) ±95% HDIs (lines) are displayed above the distributions. Gray datapaths show prior distributions for the parameters (note, the *y*-axis scaling compresses the priors for `*A*_rew_ `*A*_pun_, & *βb*). Datapoints indicate densities at *γ*_1_ = 0, from which the Bayes factors (BF_10_) were calculated.

Table 2 shows summary statistics of the posterior distributions and Figure 3 shows the posterior densities of the *γ*_1_ parameters from the joint model which capture the cross-task associations between log *k* values and the corresponding ORL parameter. The model provided very strong evidence that steep discounting was associated with lower punishment learning rates, with a 95% highest density interval (HDI) for $\gamma_{{\;}^{'}A_{\text{pen}}1}$ that *did not* overlap with 0 (Table 2) and a Bayes factor over 100. The model provided moderate evidence for an association between log *k* values and win/loss frequency sensitivities, with a 95% HDI for $\gamma_{\beta f1}$ that did not overlap with 0 and a Bayes factor of 9.95. The model showed limited evidence for an association between log *k* values and response biases, with a 95% HDI for *γ_βb_*_1_ that did not overlap with 0 but with a Bayes factor of 1.17, yielding inconclusive evidence for this association. Finally, although Figure 2 shows that higher reward learning rates were also associated with lower log *k* values, the model suggested strong evidence of no association between these parameters. The 95% HDI for $\gamma_{{\;}^{'}A_{\text{rew}}1}$ overlapped with 0 and the Bayes factor was 0.08 (or BF_01_ = 11.99 in favor of *γ*_1_ = 0). Thus, steep discounting was generally associated with lower ORL parameters, but the joint model only provided evidence that discounting was associated with punishment learning rates and win/loss frequency sensitivities, and to some degree with response biases.

### Behavioral Analyses

**Figure 4:**
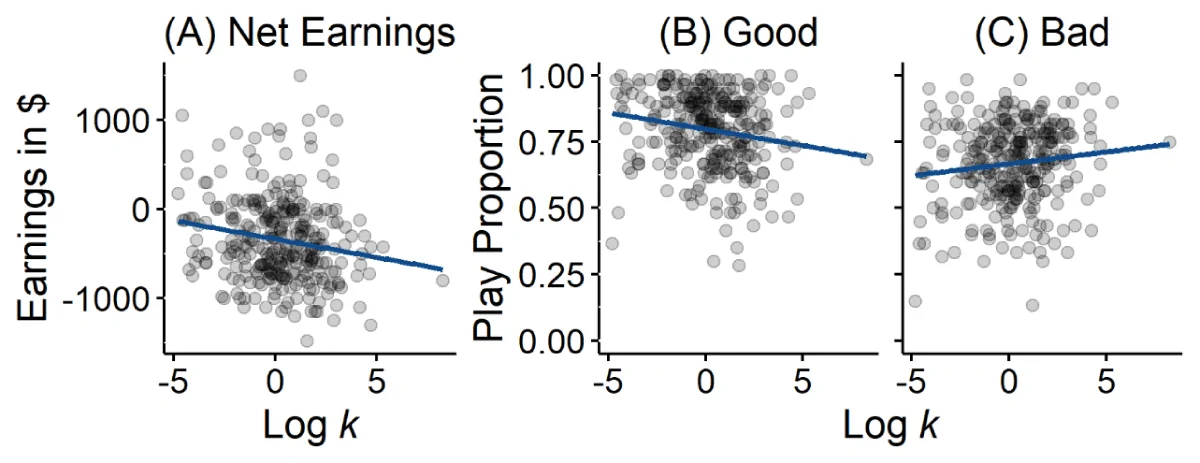
Associations Between Summary IGT Measures & Discounting. *Note*. Summary measures of IGT performance as a function of log *k* values for individual participants (datapoints) with lines of best fit.

We conducted follow-up analyses to systematically explore the associations described above. Figure 4 shows summary measures of IGT performance as a function of log *k* values. First, net earnings were negatively correlated with log *k* values (Figure 4A); that is, steep discounting (higher log *k*) was associated with lower earnings (i.e., worse performance) on the task, *r* = –.18, *p* < .01. This is consistent with the associations between log *k* values and the ORL parameters because we also found that participants with higher punishment learning rates and win/loss frequency sensitivities earned more on the IGT, `*A_pun_ r* = .30, *p* < .001, *βf r* = .33, *p* < .001. Reward learning rates (`*A_rew_*) and response biases were not associated with earnings, both *r*s ≤ |.03|, *p* > .50. Thus, steep discounting was generally associated with worse performance on the play-or-pass IGT, and this worse performance can be characterized by lower punishment learning rates and lower win/loss frequency sensitivities.

**Figure 5:**
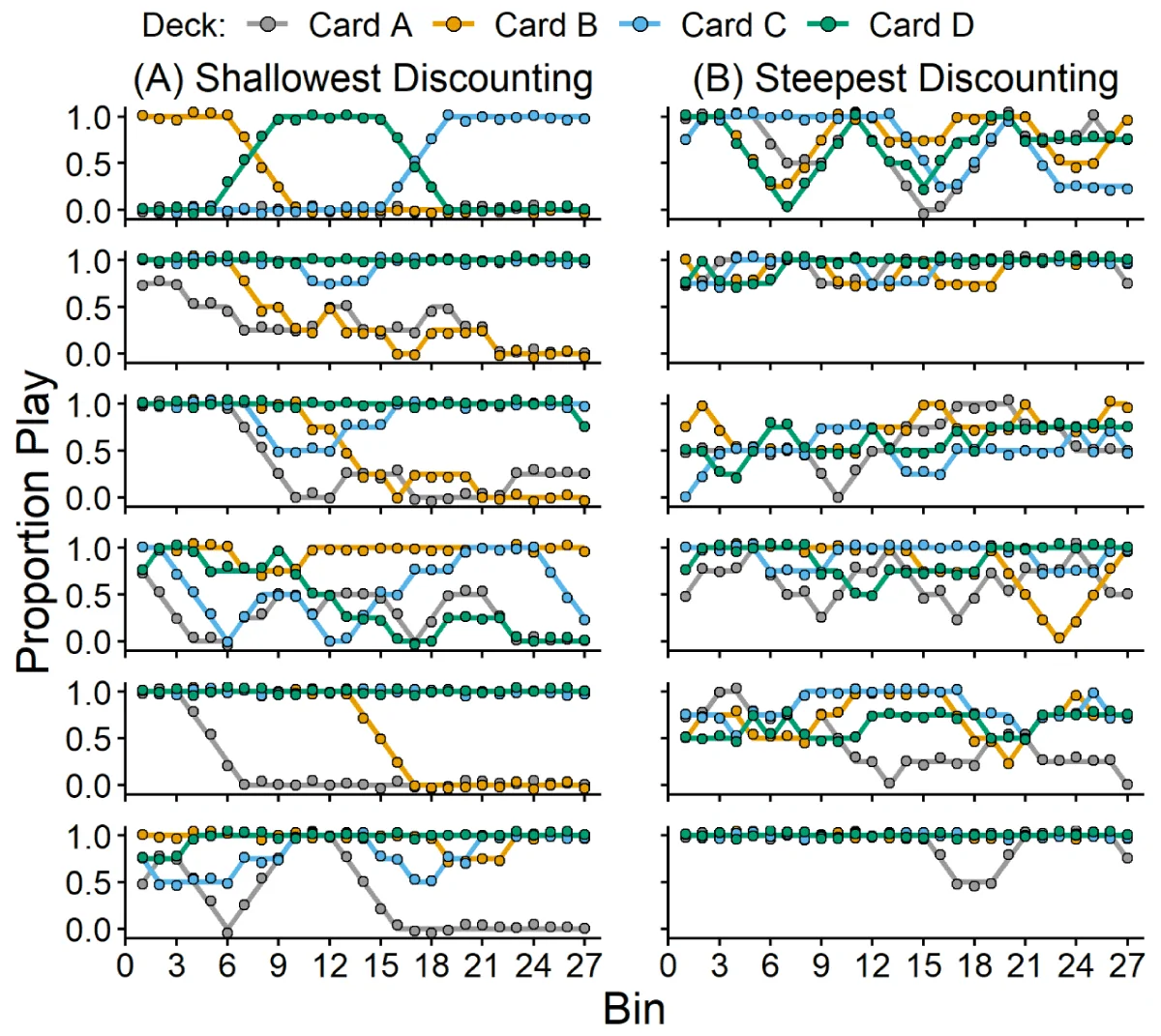
Individual Participant IGT Data. *Note*. Observed data from the play-or-pass IGT for 12 participants representing those who showed the shallowest **(A)** and steepest **(B)** discounting. Data represent choice proportions across rolling 4-trial bins. Data are jittered to show overlapping datapoints.

Second, given the task structure, we assessed associations between log *k* values and how frequently participants played on the good and bad decks separately across the whole task, calculated as play proportions. As Figure 4B and 4C show, associations between play proportions and log *k* values were weak. Playing more often on the good decks was significantly correlated with lower log *k* values, *r_S_* = –.21, *p* < .001, but playing on the bad decks was not significantly correlated with log *k* values, *r_S_* = .10, *p* = .08. This result is a bit counterintuitive given the findings above showing that lower punishment learning rates were associated with higher log *k* values. It is worth noting that punishment learning rates capture how people weight trial-level losses in the task and thus may not be well-reflected by session-wide play proportions. Thus, we also examined whether *changes* in performance across the IGT were associated with discounting. To do this, we calculated good/bad play proportions by 30-trial blocks (cf. Case & Olino, 2020) and computed difference scores between blocks 1 and 3, characterizing how participants shifted from playing at the beginning of the task to the end of the task. Difference scores were significantly correlated with log *k* values for the bad decks, *r_S_* = .21, *p* < .001, but not the good decks, *r_S_* = –.07, *p* = .24. To contextualize this finding, it can be helpful to examine individual participant data. Figure 5 shows data from 12 participants, six who showed the shallowest discounting (i.e., the 6 lowest log *k* values; Figure 5A) and six who showed the steepest discounting (i.e., the 6 highest log *k* values; Figure 5B). A notable difference between these participants is that those who showed more shallow discounting generally show a more rapid avoidance of the bad decks (i.e., passing on Decks A & B). Taken together, these findings suggest that participants who show shallow discounting learn which decks are bad more rapidly than participants who show steep discounting.

Finally, the model showed inconclusive evidence that steep discounting was associated with lower response bias. The 95% HDI for *γβ*b1 did not overlap with 0, but the Bayes factor was close to 1.0. To probe this association with the behavioral data, we correlated overall play proportions (i.e., calculated across decks), characterizing the general tendency to play, with point estimates of the log *k* values. This revealed no association between overall play proportions and log *k* values, *r_S_* = –.04, *p* = .49, suggesting that the general tendency to play on the IGT, reflected by response bias (i.e., *βb*), is not associated with delay discounting. Thus, although further research may be necessary to provide decisive evidence regarding the association between response bias on the IGT and delay discounting, our behavioral data suggest little association between response bias on the IGT and delay discounting.

### Sensitivity Analyses

One participant had a particularly high log *k* value (see datapoint on righthand side of Figure 2) which could be concerning if that participant’s data were responsible for the associations between parameters from the joint model. To ensure our results were not driven by this participant’s data, we fit the joint model without the participant. The results were qualitatively the same without this participant, with evidence in favor of higher log *k* values being associated with lower punishment learning rates, win/loss frequency sensitivities, and response biases (see supplement for details). Thus, although one participant appears to be an outlier in our data, inclusion of this participant does not change our overall findings.

In addition, to determine whether our findings would hold in a model without a joint probabilistic structure, we also examined associations between ORL parameters and log *k* values estimated via non-coupled models. That is, we estimated ORL parameters and log *k* values in separate models, then correlated parameters from each model. Details of these models are shown in the supplement, but in brief, associations between ORL parameters and log *k* values were weaker with the non-coupled models. However, overall, we replicate the main findings such that steeper discounting (higher log *k*) is associated with a lower punishment learning rate and lower win/loss frequency sensitivity.

The data consisted of some participants who were part of a mother-father dyad which arguably violates the assumption of independence between observations. Correlations between parameters from mother-father dyads were low-to-moderate (all *r*-values < |.40|) and may not be considered too problematic from a collinearity perspective (Dormann et al., 2013). However, to be conservative, we repeated our analysis with the removal of one participant from each mother-father dyad to assess whether the findings would remain. This analysis replicated the association between steep discounting and lower punishment learning rates: posterior mean $\gamma_{{\;}^{'}A_{\text{pen}}1}$ = –.05, 95% HDI [–.08, –.02]. The analysis did not replicate the association between steep discounting and win/loss frequency sensitivities: posterior mean *γ_βf_*_1_ = –.23, 95% HDI [–.48,.02]. Similar to the model fit to the full dataset, there was little association between delay discounting and response bias and reward learning rates (see supplement for further details).

**Figure 6:**
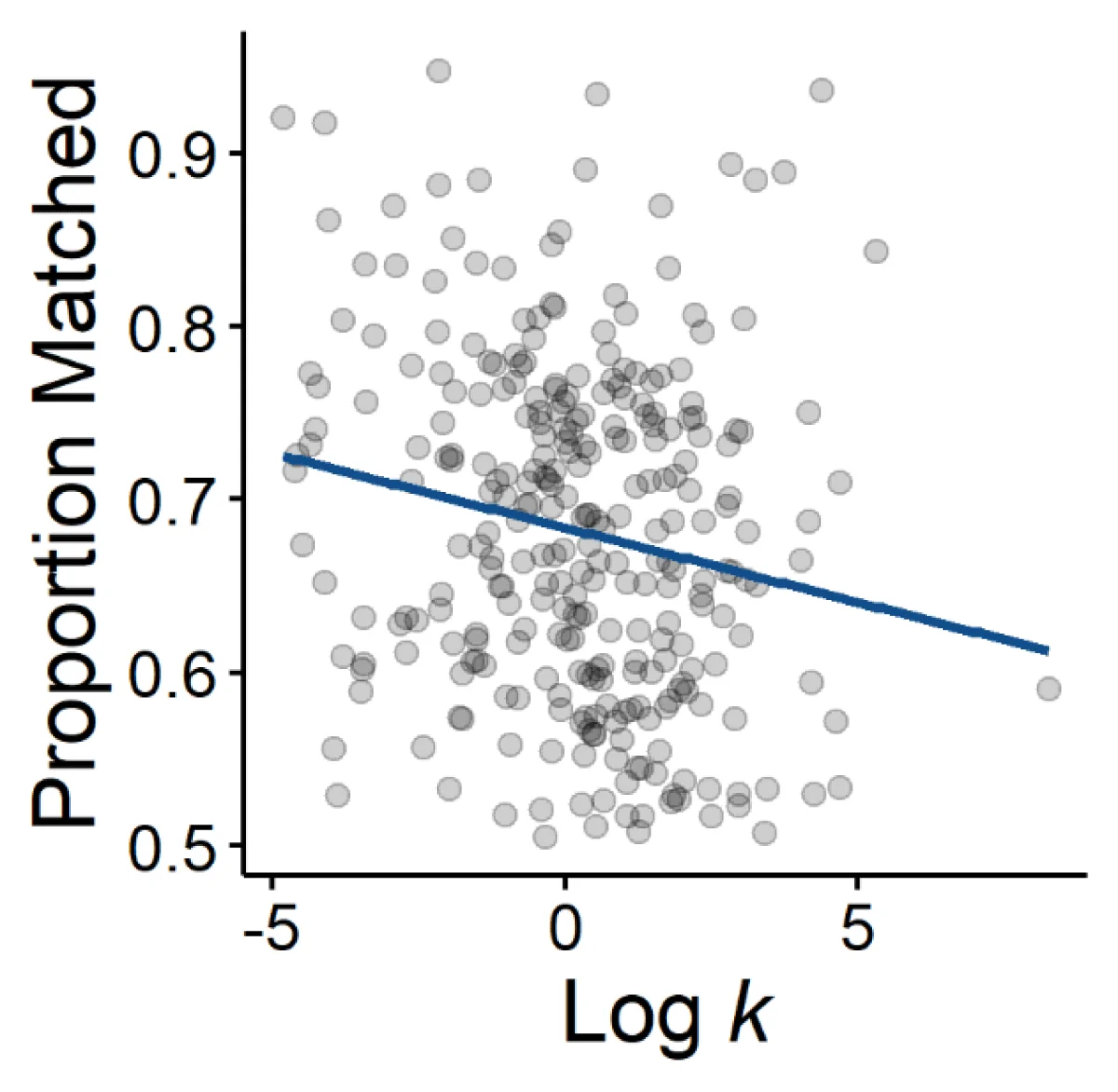
Association Between Log k & Proportion Matched. *Note*. Proportion of predicted choices matching observed choices from the IGT (Proportion Matched) as a function of log *k* values.

Finally, a helpful reviewer pointed out that participants with steep discounting may be insensitive to feedback more broadly speaking, instead of insensitive to losses as our results suggest. Our finding that participants who demonstrated steep discounting also show little change in playing on the bad decks across the task supports this possibility. Another implication of this suggestion is that participants who show steep discounting may also be more difficult for the ORL model to characterize. To explore this, we assessed whether log *k* values were associated with how well the joint model predicted individual participants’ data from the play-or-pass IGT. For this, we calculated the proportion of predicted choices matching the observed choices on the IGT (labeled “proportion matched”), using the 16000 samples from the posterior predictive checks. Next, we correlated proportion matched with log *k* values. As illustrated in Figure 6, the association between proportion matched and log *k* values was weak; however, the correlation was statistically significant, *r_S_* = –.19, *p* = .001, suggesting that the model predicted the IGT choices of participants with steep discounting less accurately.

## Discussion

The goal of this study was to examine associations between sensitivity to delayed consequences, measured by delay discounting, and computational measures of reward and punishment learning on the play-or-pass Iowa Gambling Task (IGT). Participants completed an intertemporal choice (ITC) task and the play-or-pass IGT, then we fit a joint model to simultaneously estimate parameters from a delay discounting model (for the ITC task data) and the Outcome Representation Learning Model (ORL; for the play-or-pass IGT data), as well as the associations between parameters from each model. In line with prior research, we found that steep discounting was associated with worse overall performance on the IGT in terms of earning less on the task as well as playing more often on the bad decks across the task and less often on the good decks overall (He et al., 2016; Monterosso et al., 2001; Olson et al., 2007; Wölfling et al., 2020). With the joint model, we found that steep discounting was associated with lower punishment learning rates and win/loss frequency sensitivities. The model suggested inconclusive evidence regarding the association between discounting and response bias; however, the behavioral data suggested little-to-no association exists between these parameters. Finally, although lower reward learning rates tended to be associated with steeper discounting (see Figure 2), the model provided evidence in favor of no association between these parameters (see Figure 3). We discuss these findings in further detail below.

Our study was based on the premise that how people value immediate and delayed consequences, measured by delay discounting, should be associated with patterns of decision-making for options that vary in their short- and long-term consequences. The IGT is an ideal task to measure these patterns by arranging advantageous decks, consisting of small trial-level wins that accumulate over time to produce net gains, and disadvantageous decks, consisting of large trial-level gains that are offset by losses to produce net losses in the long-term (Bechara et al., 1994). Our findings suggest that the association between steep discounting and suboptimal IGT performance reflects an insensitivity to losses in terms of both magnitude and frequency. Specifically, a lower punishment learning rate characterizes reduced sensitivity to the large losses on Deck B, and a lower win/loss frequency sensitivity characterizes reduced sensitivity to the frequent losses on Deck A (see simulation study in supplement for illustration of performance on the IGT according to low *A*_pun_ & *βf*). This may be helpful in understanding maladaptive behavior in certain forms of psychopathology, namely substance abuse. For example, Bickel et al. (2012b) have suggested that people who do not value delayed consequences, measured with steep discounting, are biased towards the short-term value of harmful reinforcers while neglecting the long-term negative consequences of those reinforcers. Generally, the finding that worse IGT performance is associated with steep discounting supports this perspective; however, our data suggest this may be due to a specific insensitivity to the magnitude and frequency of losses. Thus, it is possible that populations of people who show steep discounting (e.g., populations engaging in substance abuse) may be insensitive to the losses occurring over time that ultimately lead to larger negative consequences experienced later (e.g., incarceration, loss of social reinforcers with excessive drug use; Ignaszewski, 2021).

An alternative interpretation of our data could be that people who show steep discounting may be generally insensitive to feedback on the IGT. Their behavior was also less well captured by the model, which may reflect random responding or the use of an alternative strategy not represented by the model. In line with this possibility, we found that people who show steep discounting have lower win/loss frequency sensitivities, punishment learning rates, and although not statistically supported, lower reward learning rates (see Figure 2). Steep discounting was also associated with lower predictive performance on the IGT (*r* = –.19 between proportion matched & log *k*s) as well as less change in playing on the bad decks between the first and last blocks of the IGT (i.e., the difference score correlations). One method of testing whether steep discounting specifically explains insensitivity to the long-term losses on the IGT could be to assess interrelations between punishment learning on the IGT and discounting of delayed losses (Green et al., 2014). That is, we would expect steep discounting of losses to be more strongly associated with insensitivity to long-term losses on the IGT over and above steep discounting of gains. This is an important avenue for future research to explore, not just for more precisely identifying how discounting relates to learning (i.e., reduced punishment learning or insensitivity to feedback in general), but also in identifying alternative measures (i.e., measures of loss discounting) that provide better convergence with IGT performance.

In addition to the findings noted above, our results suggest that delay discounting is not associated with response bias and reward learning rates on the IGT. Although further research may be needed to provide decisive evidence regarding the association between discounting and response bias (cf. the BF_10_ = 1.17), the lack of association between these parameters may not be surprising given that response bias characterizes a general tendency to play which may not have a strong theoretical interpretation (although see Haynes et al., 2025 for possible interpretations). The lack of association between delay discounting and reward learning rates, however, may be surprising because the intertemporal choice task involved choices about monetary rewards which, at face value, would be expected to relate to reward learning. This finding may be due to reward learning rates being difficult to estimate from the ORL model, reflected by the suboptimal recovery of these learning rates (*r* = .48, see supplement). Although adjusting the structure of the play-or-pass ORL could improve estimation of reward learning rates, our lab has generally found that alternative formulations of the ORL perform worse (see supplement & Haynes et al., 2024). Instead, reward learning rates may be specifically difficult to estimate from this version of the IGT because of the typical patterns of behavior we observe on the task. Participants typically play frequently at the beginning of the task, likely to gain information about the decks (see also Haynes et al., 2025). Across trials, participants adjust their behavior in response to the losses from the decks which would primarily be reflected by the punishment learning rates. Thus, we advise researchers specifically interested in how *reward valuation*, measured with delay discounting, and *reward learning*, measured with IGT-like decision-making tasks, against using the play-or-pass IGT. Alternative tasks, like the traditional IGT may be better suited for studying these associations, although further research would be necessary to confirm this possibility.

### Future Directions

Overall, our data suggest several avenues for future research. First, given that steep delay discounting and dysfunctional reward and punishment learning are associated with psychopathology (e.g., substance abuse; MacKillop et al., 2011; Mukherjee & Kable, 2014), identifying how these constructs uniquely predict different forms of psychopathology is an important next step. For example, research employing both an intertemporal choice task and the IGT may be able to use computational metrics (e.g., log *k* values & *A_pun_*) to distinguish risk for different forms of psychopathology (e.g., depression & substance abuse). This is particularly important for using delay discounting as a tool to measure predisposition towards psychopathology. Because steep discounting predicts such a wide range of disorders (Bickel et al., 2012a, 2019), it tells little about what disorder(s) an individual may be predisposed to (Bailey et al., 2021). Therefore, employing multi-faceted assessments, like intertemporal choice tasks and the Iowa Gambling Task, may improve the precision with which we can predict specific forms of psychopathology.

Second, it will be important to examine whether variables known to affect delay discounting also affect reward and punishment learning on the IGT (& vice versa) in predicted patterns. For example, people reliably show steeper discounting for smaller delayed rewards relative to larger delayed rewards, known as the magnitude effect (Green et al., 1997, 2013). If delay discounting contributes to IGT performance as shown above, then increasing gain/loss magnitude on the IGT should result in better performance, reflected by higher punishment learning rates and higher win/loss frequency sensitivities. Furthermore, given the overlap in neural systems involved in delay discounting and IGT performance (e.g., ventromedial prefrontal cortex; Bechara et al., 1994; Fellows & Farah, 2005; Mukherjee & Kable, 2014), studies could explore how changes in task performance impact neural function to provide a more mechanistic understanding of these overlapping systems. These studies would be important for providing further evidence illustrating the relation between delay discounting and reward and punishment learning that could have practical implications. For example, interventions that reduce steep discounting (i.e., reduce impulsive decision-making) could also be promising interventions or preventions for improving dysfunctions in reward and punishment learning (e.g., Rung et al., 2019; Rung & Madden, 2018).

Finally, further research is necessary to precisely identify how delay discounting and reward and punishment learning are related. Our data suggest that steep discounting is associated with a lower sensitivity to punishment via a lower punishment learning rate and lower win/loss frequency sensitivity, but as discussed above, this could reflect an insensitivity to feedback more broadly. Because lower punishment learning rates and win/loss frequency sensitivities are specifically associated with worse performance on the play-or-pass IGT (i.e., playing more on bad decks), we may be capturing a tendency for steep discounting being associated with suboptimal decision-making more generally. For example, if we arranged a gambling task in which lower punishment learning rates and win/loss frequency sensitivities were associated with better performance, would people who show steep discounting perform better or worse on the gambling task? At face value, our data predict the former; that is, steep discounting being associated with better IGT performance. Such a finding would connect to literature suggesting that what we label as “impulsive” or “suboptimal” is contextually dependent. For example, seemingly impulsive behavior like steep discounting is adaptive in environments where the likelihood of attaining a delayed outcome is lower (e.g., areas with high mortality rates & lower life expectancies; Daly & Wilson, 2005; Stevens & Stephens, 2010). Connecting to this literature could be helpful in understanding why steep discounting develops in the first place (i.e., as an adaptive response to the environment). Thus, identifying how delay discounting relates to (sub)optimal performance on tasks measuring reward and punishment learning, like the IGT, could help us identify the contexts that favor steep discounting.

An alternative method of testing the specific relation between delay discounting and reward and punishment learning could involve using additional tasks to assess convergence of associations. This study tested performance on two tasks; however, additional tasks could be used that assess similar constructs and that have similar computational parameters (Ahn et al., 2017). With more than two tasks, more complex models, including latent variable models with parameters reflecting the same underlying constructs could be used to assess overlap in these processes, as well as predict clinical outcomes (Cooper et al., 2019). Importantly, our study, and these extensions, could enhance the predictive utility of these tasks beyond examining individual parameters from a single task (Cuthbert & Insel, 2013; Morris et al., 2022).

### Strengths & Limitations

This study has multiple strengths. First, we assessed cross-task associations using a joint modeling framework to more adequately measure how performance on the intertemporal choice task related to performance on the Iowa Gambling Task. The joint modeling framework allowed us to account for uncertainty in estimating parameters across models that otherwise would have attenuated the associations between the two tasks (see non-coupled model in supplement; Haines et al., 2023, 2025). Second, and related to the previous strength, we used a computational approach to model IGT performance which provided metrics that characterize trial-level performance on the task. Although much can be learned from traditional scoring measures like the proportion of good/bad plays (Case & Olino, 2020; Cauffman et al., 2010), these measures cannot account for key patterns of behavior on the IGT such as sensitivity to win/loss frequency (Chiu et al., 2008; Steingroever et al., 2013). As our data suggest, win/loss frequency sensitivity is an important aspect of IGT performance related to delay discounting. Likewise, the ORL model showed that punishment learning is associated with delay discounting, pointing to an important decision-making process (e.g., punishment sensitivity) that would not have been captured by traditional scoring measures (e.g., net score; Bechara, 2007). Finally, our results are supported by individual participant data (see Figure 5), particularly with respect to the elevated punishment learning rates and win/loss frequency sensitivities. Computational methods are helpful in distinguishing the multiple behavioral processes that contribute to task performance; however, because of the complexity of the models, and the theoretical implications of those models (e.g., measuring unobservable constructs), tying those behavioral processes back to the observable patterns of behavior provides further confidence in the findings from those models (Wilson & Collins, 2019).

In spite of these strengths, this study also has weaknesses. We used a simple delay discounting model to characterize discounting, but alternative models could be used to measure other decision-making processes on intertemporal choice tasks. We chose the hyperbolic discounting model because of its common use (e.g., Fontes et al., 2025; Klein et al., 2022; Kohler et al., 2022) and because this was the model used by Kvam et al. (2021) from whom we based on our joint modeling approach on. In addition, our model-based results were inconclusive regarding the association between delay discounting and response bias from the IGT. Though our behavioral data suggests no correlation between response bias and discounting, further research may be necessary to provide decisive evidence in favor of (or against) an association between these measures. Similarly, although we found moderate evidence of an association between win/loss frequency sensitivities and log *k* values, this association was weak (*r* = –.21; see Figure 2 & Table 2) and did not remain in one of our sensitivity analyses (i.e., when removing one participant from each dyad); therefore, replication will be important to ensure this effect is reliably demonstrated.

Additionally, the study consisted of parents from a project focused on the Positive and Negative Valence Systems in children and adolescents which poses two potential issues. First, some participants were part of mother-father dyads which, as noted above, may violate the assumption of independence between observations. Although the correlations between parameters suggest that non-independence may not be problematic, when we take a conservative approach by removing one participant from each dyad, our results are weakened. Second, the sample was composed primarily of mothers (see Table 1). Therefore, our results may not generalize to populations of people without children or populations with a more balanced distribution of male and female participants. Thus, replication is vital to validate our findings, particularly in samples wherein participants are independent and that are drawn from a broader, more balanced, population.

Finally, we did not assess the reliability of measures from either task. Prior research indicates that measures of delay discounting show strong rank-order stability across time (*r* = .81, Gelino et al., 2024; see also Miller et al., 2023) and generalize across different types of outcomes (Odum et al., 2020), suggesting that reliability of our discounting measure may be of little concern. In contrast, measures from the Iowa Gambling Task (IGT) are mixed in terms of rank-order consistency across time (Buelow & Barnhart, 2018; Schmitz et al., 2020) and reinforcement learning (RL) parameters are known to have poor reliability and generalizability (Eckstein et al., 2022; Karvelis et al., 2023), suggesting that our findings may not generalize beyond the present study, particularly with different IGT-like tasks. Haynes et al. (2024) assessed test-retest reliability of measures from the same play-or-pass IGT used in the present study but in college students, with some promising findings (*r*-values ≥ .49 for summary measures, *r*-values ≥ .59 for ORL parameters). However, although significant, they found that rank-order stability degrades substantially when ORL parameters are estimated without a joint probabilistic structure defining parameters across two sessions (*r*-values = .21–.35; see their supplement). Joint modeling across tasks, as in this study, should improve parameter reliability (Haines et al., 2025), but we urge caution when generalizing beyond this study, particularly with respect to measuring RDoC constructs in the Positive and Negative Valence Systems (e.g., punishment learning), as differences across time or tasks (e.g., different RL tasks) may not replicate.

## Conclusion

In conclusion, this study provides important insights into how decision-making across two RDoC constructs, reward valuation and reward and punishment learning, relate to one another. We found that delay discounting, a common measure of reward valuation, is associated with multiple processes characterizing reward and punishment learning on the play-or-pass IGT. These findings have both theoretical and practical implications that require further testing but may prove useful in understanding how reward valuation and reward and punishment learning contribute to mental illness.

## Additional File

The additional file for this article can be found as follows:

10.5334/cpsy.172.s1 Supplement. Supplementary file contains additional model information and sensitivity analyses.

## Data Availability

Data and code are publicly available on the Open Science Framework (OSF) at https://osf.io/rsqx7/?view_only=26ade4cbcba64c83bfb9c6f07f88c650.

## Footnotes

As a comparison, the original IGT involves presenting decks simultaneously such that playing on one deck is mutually exclusive from playing on the other decks. Because of this, standard scoring procedures for the original IGT cannot differentiate whether shifts in playing on the good decks reflect reward learning (i.e., approaching the good decks), punishment learning (i.e., avoiding the bad decks), or both.

It should be noted that these posterior *p*-values summarize across all data and thus do not provide detail regarding specific points that the model tends to over/underestimate. For example, As Figure 1B shows, the model tends to underestimate indifference points at the longest delay which has been previously reported with the hyperbolic discounting model (McKerchar et al., 2009).
