# Supplementary material for: Associations Between Steep Delay Discounting and Punishment Learning
Source: Comput Psychiatr. 2026 Sep 1;10(1):159–83. doi: 10.5334/cpsy.172 (PMC13544050; doi:10.5334/cpsy.172)
Supplement: Supplementary file 1 [file cpsy-10-1-172-s1.pdf]

Supplement

This file is the supplement to the manuscript titled Associations Between Steep Delay Discounting and Punishment Learning. Models presented in this supplement were fit using the same general procedures described in the main text, unless otherwise noted. Specifically, we used a hierarchical Bayesian approach to fit the models in Stan (v. 2.36.0; Stan Development Team, 2023) with R (v. 4.4.3; R Core Team, 2025) as the interface with Stan via the CmdStanR package (v. 0.9.0; Gabry et al., 2025). Person level parameters were drawn from group-level distributions to leverage hierarchical pooling. We sampled the models using 4 chains with 5000 iterations each (1000 warmup & 4000 target samples). For each model, we conducted typical diagnostic assessments: evaluating trace-plots and $\hat{R}$ values to assess chain convergence (Gelman & Rubin, 1992). All models presented below showed convergence.

ORL Simulation Study

We conducted a simulation study to illustrate how different parameter values from the Outcome Representation Learning (ORL) model translate to patterns of behavior on the play-or-pass Iowa Gambling Task (IGT). To do this, we simulated choice data from the ORL based on point estimates (posterior means) from individual participants from the joint model. For each parameter, we chose the lowest, median, and highest point estimate to represent low, moderate, and high parameter values, respectively. For simplicity, we simulated choice data by varying levels of one parameter while maintaining a moderate value for the other parameters. For example, simulations for reward learning rates (A_{rew}) involved simulating data with a high, moderate, and low value for A_{rew} and moderate values for A_{pun}, □f, and □b. Parameter values used in the simulations are displayed in Table S1. For each simulation, we simulated choice data from 100 hypothetical participants.

Table S1
ORL Simulation Parameters

Level	A_{rew}	A_{pun}	□f	□b
Low	.0454	.0176	-3.4914	-1.0511
Moderate	.1146	.0718	3.3194	0.9931
High	.2289	.4298	13.9692	2.8164

Note. Parameter values, rounded to 4 decimal places, used in simulation study.

After simulating data, we calculated play proportions across the 100 hypothetical participants for each simulation. Play proportions are depicted by trial for each deck in Figure S1. Overall, the results of the simulations are consistent with the finding that elevated punishment learning rates and win/loss frequency sensitivities are associated with better performance on the task, noted in the main text. Specifically, high levels of these parameters are associated with a more rapid decline in playing on the bad decks (A & B; see middle two panels). High punishment learning rates (A_{pun}) are associated with a decline in playing on both decks A and B; however, choices for deck B decline more sharply. Deck B is

associated with the highest single-trial loss which would be weighted highly when punishment learning rates are high. High win/loss frequency sensitivities are associated with playing less on deck A primarily. This is important for validity purposes as deck A has the highest frequency of losses in the task (see Table S2 for task descriptive statistics); therefore, the win/loss frequency sensitivity parameter appears to capture sensitivity to these more frequent losses.

Figure S1

Simulated Data from the ORL

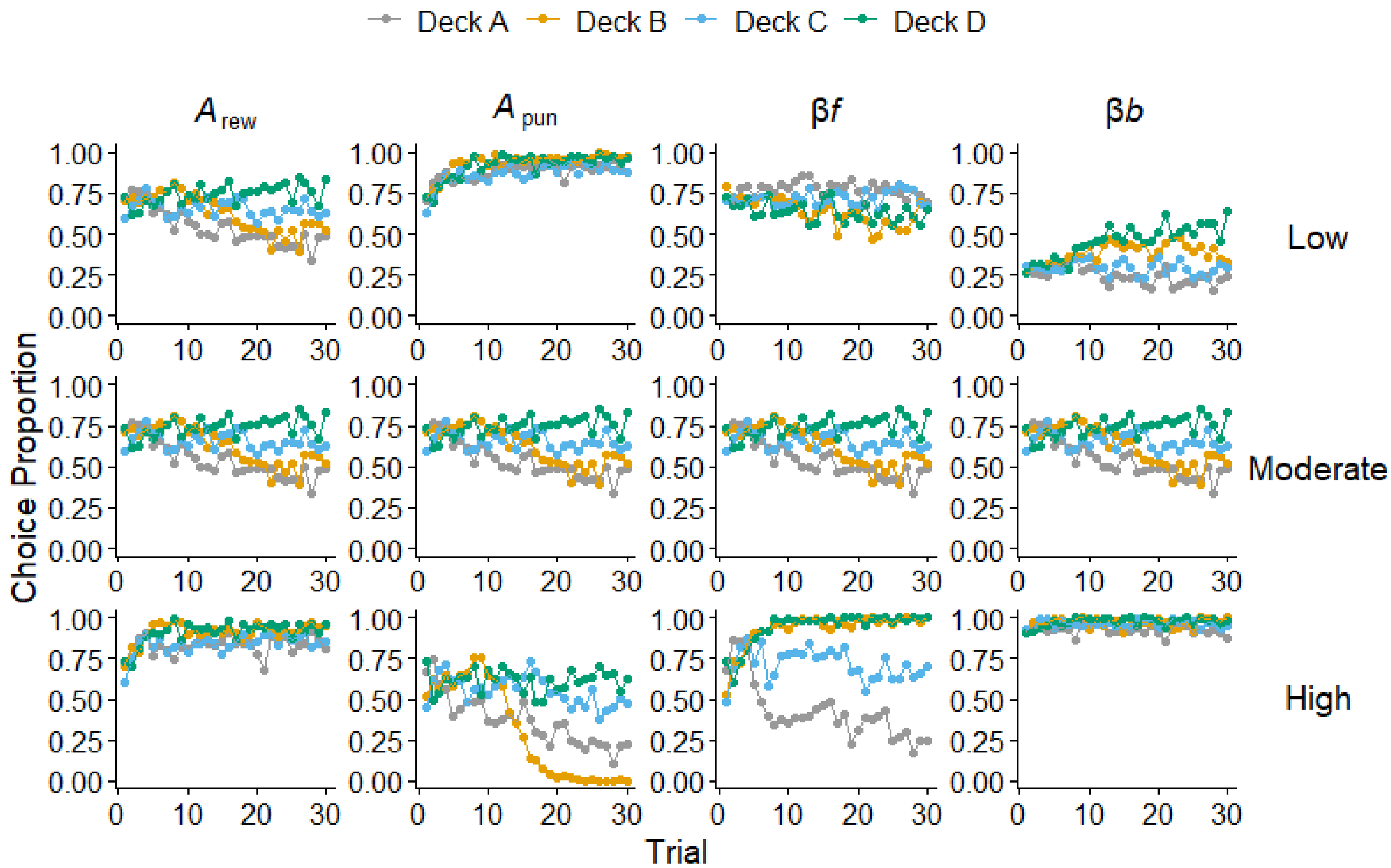

Note. Simulated play proportions across trials for each deck according to varying ORL parameter values (see Table S1).

Table S2

Task-Wide Descriptive Statistics

Statistic	A	B	C	D
Mean	-25	-25	20	25
Sum	-750	-750	600	750
Win Frequency	15	27	24	27
Loss Frequency	15	3	6	3
Highest Gain	100	100	50	50
Highest Loss	-250	-1150	-25	-200

Note. Descriptive statistics for the outcomes in the play-or-pass IGT. Win frequency includes both positive outcomes (x(t) > \$0) and neutral outcomes

(x(t) = 0).

ORL Parameter Recovery

A common method of assessing bias in a model is to conduct parameter recovery diagnostics (Wilson & Collins, 2019). In brief, parameter recovery involves simulating data from a model according to known parameter values and then fitting the model to those data to assess how well the model "recovers" the parameters. To do this, we first calculated posterior means of the person-level parameters from the joint model presented in the main text. Next, we simulated data (IGT choices & indifference points) from those parameters. Finally, we fit the joint model to the simulated data and assessed parameter recovery by (1) correlating "true" parameters with "recovered" parameters and (2) examining whether recovered parameters replicated the cross-tasks associations presented in the main text.

In general, parameters were recovered well from the joint model. Figure S2 shows posterior means of the recovered parameters as a function of the posterior means of the true parameters for individual participants. Correlations between recovered and true parameters were all in the moderate to strong range. Reward learning rates showed the poorest parameter recovery which has been previously reported with this model (Haynes et al., 2024) and may suggest this parameter is difficult to estimate.

Figure S2

Parameter Recovery for Joint Model

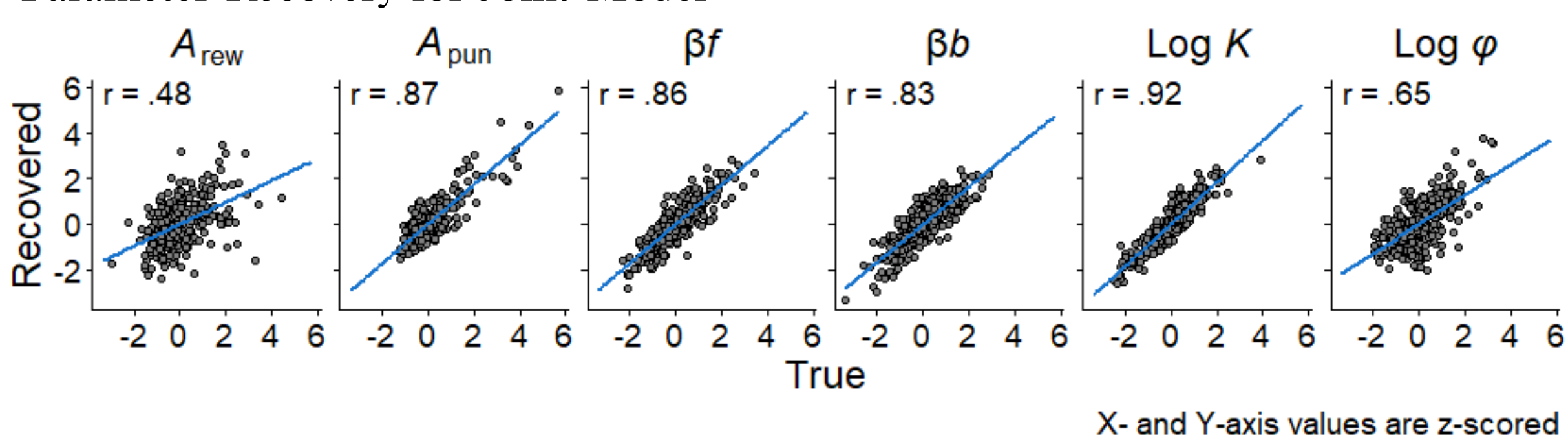

Note. Recovered parameters as a function of the actual parameters from the ORL model. We transformed parameters to z-scores for visualization purposes.

Recovered parameters also replicated the cross-task associations presented in the main text. Specifically, recovered parameters showed that that steep discounting (higher log k) was associated with lower punishment learning rates ($\gamma_{A_{pun}1}$ 95% HDI [-0.12,-0.07]), lower win/loss frequency sensitivities ($\gamma_{\beta f1}$ 95% HDI [-0.62,0.16]), and lower response biases ($\gamma_{\beta b1}$ 95% HDI [-0.16,-0.06]). In sum, parameters from the joint model can be recovered well for individual participants and replicate the main findings from the text.

Comparison of the ORL Model with Standard RL Models

As noted in the main text, we examined whether the Outcome Representation Learning (ORL) model outperforms simpler reinforcement learning (RL) models. As with the ORL model, these simpler RL models modeled choices to play according to Bernoulli likelihood functions (Equation 4 from the main text re-presented below):

$$Y_{i,j}(t) \sim \text{Bernoulli}\left(\frac{1}{1 + exp(-V_{i,j}(t))}\right)$$

Equation S1

where $Y_{i,j}(t)$ is the ith participant's choice to play (Y = 1) or pass (Y = 0) on the jth deck on trial t and $V_{i,j}(t)$ is the value of playing when presented with deck j on trial t by participant i. For both RL models, value (V) updated from trial to trial using a reinforcement learning algorithm:

$$V_{i,j}(t+1) = EV_{i,j}(t+1) + \beta b_i$$

Equation S2

where $EV_{i,j}(t+1)$ is the updated expected value of deck j and □ ḅ is a response bias parameter.

For one model, hereinafter referred to as the 2-parameter RL model, expected value updated according to a single learning rate as follows:

$$EV_{i,j}(t+1) = \begin{cases} EV_{i,j}(t) + A_i \cdot \left(x(t) - EV_{i,j}(t)\right) & if\ Y_{i,j}(t) = 1 \\ EV_{i,j}(t+1) = EV_{i,j}(t) & if\ Y_{i,j}(t) = 0 \end{cases}$$

Equation S3

where $EV_{i,j}(t)$ is the expected value, x(t) is the outcome (e.g., win \$200 = +100; lose \$200 = -100), A is a learning rate describing updating in response to gains (x(t) > 0), losses (x(t) < 0), and neutral outcomes (x(t) = 0).

For the other model, hereinafter referred to as the 3-parameter RL model, expected value updated according to a reward learning rate (A_{rew}) and punishment learning rate (A_{pun}):

$$EV_j(t+1) = \begin{cases} EV_j(t) + A_{rew} \cdot (x(t) - EV_j(t)) & if\ Y_j(t) = 1 \text{ and } x(t) \geq 0 \\ EV_j(t) + A_{pun} \cdot (x(t) - EV_j(t)) & if\ Y_j(t) = 1 \text{ and } x(t) < 0 \\ EV_j(t) & if\ Y_j(t) = 0 \end{cases}$$

Equation S4

where $EV_{i,j}(t)$ and x(t) are as described above, A_{rew} is a learning rate for gains or neutral outcomes (x(t) □ 0), and A_{pun} is a learning rate for losses (x(t) < 0). Note, the 3-parameter RL model is identical to the ORL, except win/loss frequency is not taken into account.

For both models, we used weakly informative priors on the group-level parameters, and the group-level parameters served as priors for the person-level parameters for

hierarchical pooling:

2-Parameter Model Group-Level Priors:

$$
\begin{aligned}
\mu'_A, \mu_{\beta b} &\sim \text{Normal}(0,1) \\
\sigma'_A &\sim \text{Half-Normal}(0.2) \\
\sigma_{\beta b} &\sim \text{Half-Normal}(1) \\
\Phi^{-1}(A_i \;/\; scale) &\sim \text{Normal}(\mu'_A, \sigma'_A) \\
\beta b_i &\sim \text{Normal}(\mu_{\beta b}, \sigma_{\beta b})
\end{aligned}
$$

3-Parameter Model Group-Level Priors:

$$
\begin{aligned}
\mu'_{A_{rew}}, \mu'_{A_{pun}}, \mu_{\beta b} &\sim \text{Normal}(0,1) \\
\sigma'_{A_{rew}}, \sigma'_{A_{pun}} &\sim \text{Half-Normal}(0.2) \\
\sigma_{\beta b} &\sim \text{Half-Normal}(1) \\
\Phi^{-1}(A_{rew,i} \;/\; scale) &\sim \text{Normal}(\mu'_{A_{rew,i}}, \sigma'_{A_{rew}}) \\
\Phi^{-1}(A_{pun,i} \;/\; scale) &\sim \text{Normal}(\mu'_{A_{pun,i}}, \sigma'_{A_{pun}}) \\
\beta b &\sim \text{Normal}(\mu_{\beta b}, \sigma_{\beta b})
\end{aligned}
$$

We conducted model-comparisons between these two simpler RL models with the ORL model. To do this, we used the leave-one-out information criterion (LOOIC) to examine how well the models predicted data one trial ahead for each participant based on their parameter values (Vehtari et al., 2017). LOOIC can be interpreted similar to deviances such that lower values indicate the better-fitting model. Overall, LOOIC showed that the ORL outperformed both models, displayed in Table S3.

Table S3
Model-Comparisons

Model	LOOIC	SE
PPORL	34795.1	195.2
3-Parameter RL	36734.5	185.7
2-Parameter RL	37074.3	186.1

Note. Leave-one-out-information-criteria (LOOIC) and standard errors (SE) of the LOOIC for the models fit to the play-or-pass IGT data. Lower LOOIC values indicate better model fit.

Adjusting-Amount Algorithm

The intertemporal choice task employed an adjusting-amount algorithm, based on Du et al. (2002), to obtain indifference points at five delays, presented in blocks of 9-11 trials each. For each delay block, the starting value of the smaller-immediate reward was $200, $500, or $800, randomly determined, and the value of the larger-delayed reward was always $1000. Across trials within a delay block, the amount of the immediate reward was increased or decreased based on participants' previous

choices. On the first trial, if the participant selected the immediate reward, then the immediate reward would decrease by one-half. For example, if the first trial of a delay block asked participants "Which would you prefer? $200 now or $1000 in 1 year", and the participant selected the $200 now, the immediate reward would decrease to $100 on the subsequent trial. If, in contrast, the participant selected the delayed reward on the first trial, then the immediate reward would increase by half the difference between the immediate and delayed rewards. Using the previous example above, if the participant instead selected the $1000 in 1 year, then the immediate reward would be $600 on the subsequent trial ([$1000-$200]/2+$200). Subsequent adjustments were half of the previous adjustment; if the participant selected the smaller-immediate reward, that reward decreased, and if the participant selected the larger-delayed reward, the smaller reward would increase.

Hyperbolic Discounting Model

Here, we describe the re-arrangement of the logistic form of the hyperbolic discounting model into a linear model. This form of the hyperbolic discounting model is based on Patt et al. (2021). As noted in the main text, the hyperbolic discounting model can be expressed as a logistic function (cf. Equation 3):

$$'V_{i,j} = \frac{1}{1 + e^{\log k_i + \log D_j}} \quad \text{Equation S5}$$

where $'V_{i,j}$ is the normalized indifference point for participant i at delay j, k is a free parameter characterizing the degree of discounting, and D is the delay. We can derive a linear model by re-arranging Equation S5 as follows:

Cross multiply Eq. S5

$$1 = {'V_{i,j}}(1 + e^{\log k_i + \log D_j})$$

Distribute $'V_{i,j}$

$$1 = {'V_{i,j}} + {'V_{i,j}} e^{\log k_i + \log D_j}$$

Subtract $'V_{i,j}$

$$1 - {'V_{i,j}} = {'V_{i,j}} e^{\log k_i + \log D_j}$$

Divide by $'V_{i,j}$

$$\frac{1 - {'V_{i,j}}}{'V_{i,j}} = e^{\log k_i + \log D_j}$$

Take the logarithm

$$\log \frac{1 - {'V_{i,j}}}{'V_{i,j}} = \log k_i + \log D_j \quad \text{Equation S6}$$

where all terms in Equation S6 (identical to Equation 3 in the main text) are as described above.

Prior Choices

We chose the priors used in the main text based on the literature and on simulations from the priors. For the linear functions connecting parameters from the hyperbolic discounting model to the parameters from the ORL model, we chose weakly informative priors on the intercept coefficients (i.e., the $\square_0$s) with a more informative set of priors on the slope coefficients (i.e., the $\square_1$s). More informative priors on the slope coefficients were chosen to improve model convergence. These stronger priors on the slope coefficients are also more conservative, such that the effects of interest (i.e., cross-task associations) are drawn closer to 0 (i.e., towards no association). For the ORL model parameters, we chose weakly informative priors based on previous research using this model (Haynes et al., 2024, 2025). Finally, we chose priors for parameters of the hyperbolic discounting model using simulations from the priors, discussed in further detail below.

For the hyperbolic discounting model, we simulated from different priors to generate plausible log k values. Figure S3 shows distributions of simulated log k values for three sets of group-level priors on log k.

Figure S3
Log k Priors

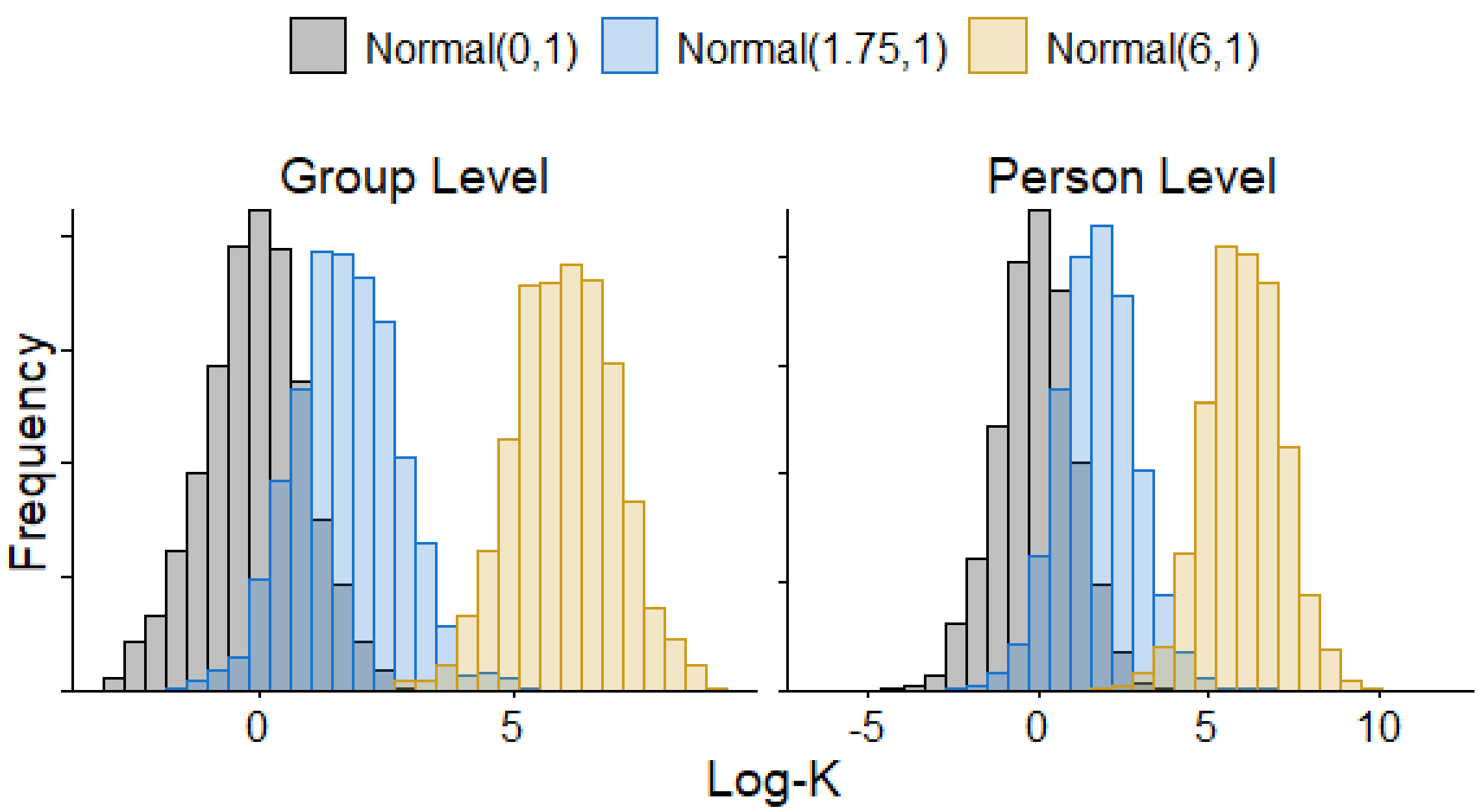

Note. Log k values simulated from three sets of prior distributions for the hyperbolic discounting model.

Each of these priors on log k imply a different pattern of indifference points which is an important consideration when fitting the hyperbolic discounting model to the indifference points. To illustrate, Figure S4 shows the implied indifference points from the person-level log k values shown in Figure S3. As figure S4 shows, selecting a different mean for the prior on the group-level log k causes the implied indifference

points across delays to vary substantially. At the extremes, a Normal(0,1) for the group-level mean log k favors higher indifference points whereas a Normal(6,1) favors lower indifference points. The Normal(1.75,1) prior tends to lie between these extremes. Thus, the choice of the prior for the group-level log k has important implications for using the hyperbolic discounting model.

Figure S4
Indifference Points from Priors

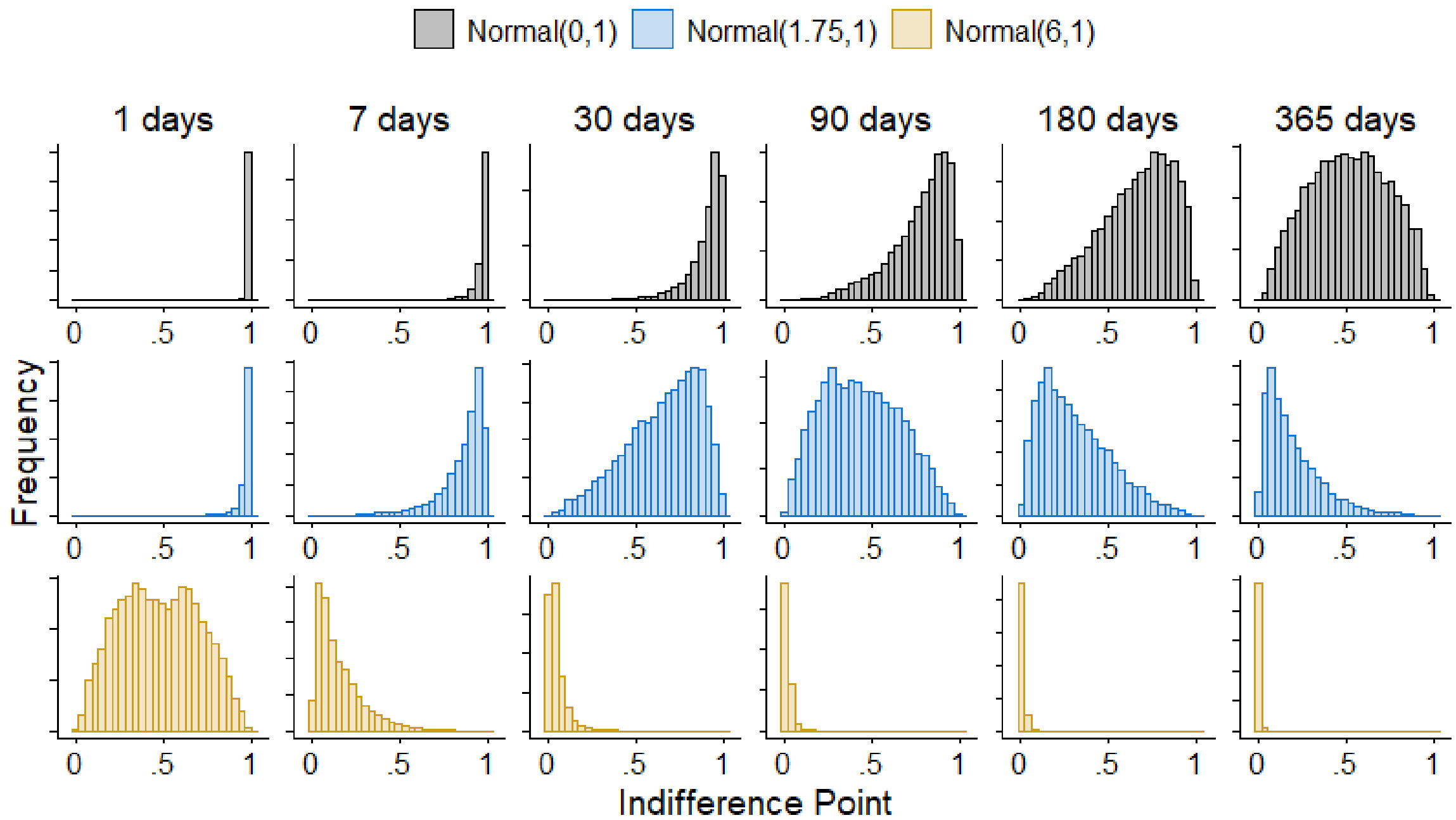

Note. Implied indifference points based on the log k priors displayed in

To address concerns regarding whether the choice of one prior over another would meaningfully influence our results, we fit the joint model using each of the priors shown above: $□_{\text{log k}}$ ~ Normal(0,1), $□_{\text{log k}}$ ~ Normal(1.75,1), and $□_{\text{log k}}$ ~ Normal(6,1). Fitting the model with each set of priors resulted in the same pattern of results. That is, steeper discounting (higher log k) was associated with lower punishment learning rates (A_{pun}), win/loss frequency sensitivities ($□_f$), and response biases ($□_b$). Descriptive statistics of the posterior distributions of the models' parameters are presented in Table S4.

Table S4
Results Using Different Priors

	Group-Level Prior		
Parameter	N(0,1)	N(1.75,1)	N(6,1)
$\gamma'_{A_{rew},0}$	-1.21 [-1.29,-1.14]	-1.21 [-1.29,-1.14]	-1.21 [-1.29,-1.14]
$\gamma'_{A_{pun},0}$	-1.42 [-1.48,-1.36]	-1.42 [-1.48,-1.36]	-1.42 [-1.48,-1.36]
$\gamma_{\beta f,0}$	3.42 [2.98,3.91]	3.42 [2.98,3.91]	3.43 [2.99,3.92]
$\gamma_{\beta b,0}$	1.05 [0.94,1.15]	1.05 [0.94,1.15]	1.05 [0.94,1.16]

	Group-Level Prior		
Parameter	N(0,1)	N(1.75,1)	N(6,1)
$\gamma_{A_{rew},1}$	-0.02 [-0.06,0.01]	-0.02 [-0.06,0.01]	-0.02 [-0.06,0.01]
$\gamma_{A_{pun},1}$	**-0.07 [-0.10,-0.04]**	**-0.07 [-0.10,-0.04]**	**-0.07 [-0.10,-0.04]**
$\gamma_{\beta f,1}$	**-0.32 [-0.54,-0.09]**	**-0.32 [-0.54,-0.09]**	**-0.32 [-0.54,-0.08]**
$\gamma_{\beta b,1}$	**-0.07 [-0.12,-0.01]**	**-0.07 [-0.12,-0.01]**	**-0.07 [-0.12,-0.01]**
	Group-Level Prior		
Parameter	N(0,1)	N(1.75,1)	N(6,1)
$\mu_{A_{rew}}$	0.12 [0.10,0.13]	0.12 [0.10,0.13]	0.12 [0.10,0.13]
$\mu_{A_{pun}}$	0.09 [0.08,0.10]	0.09 [0.08,0.10]	0.09 [0.08,0.10]
$\mu_{\beta f}$	3.36 [2.89,3.83]	3.36 [2.89,3.83]	3.33 [2.88,3.82]
$\mu_{\beta b}$	1.03 [0.92,1.14]	1.03 [0.92,1.14]	1.03 [0.92,1.14]
$\mu_{\log k}$	0.21 [-0.05,0.47]	0.21 [-0.05,0.47]	0.31 [0.05,0.56]
$\gamma_{\log \phi}$	0.58 [0.53,0.64]	0.58 [0.53,0.64]	0.58 [0.53,0.64]

Note. Posterior means (+/- 95% highest density intervals) for the models using different sets of priors. The only meaningful difference in parameter estimates is the group-level mean log k (i.e., □$_{\log k}$).

Using Indifference Points Instead of Choice Data

Bayesian methods of estimating parameters from the hyperbolic discounting model typically rely on using a Bernoulli likelihood to model trial-level choice data instead of a Normal likelihood to model indifference points. Although the Bernoulli approach allows researchers to specify weakly informative priors across all delays, unlike the Normal likelihood (see above), we found this approach estimated k-values that did not align well with the observed indifference points. To illustrate, Figure S5 shows fits of the hyperbolic discounting model using a Normal likelihood (shown in the main text) and fits using a Bernoulli likelihood. The datapoints correspond to median indifference points with interquartile ranges. The model using the Normal likelihood clearly aligns with the indifference points better.

Figure S5
Likelihood Comparisons

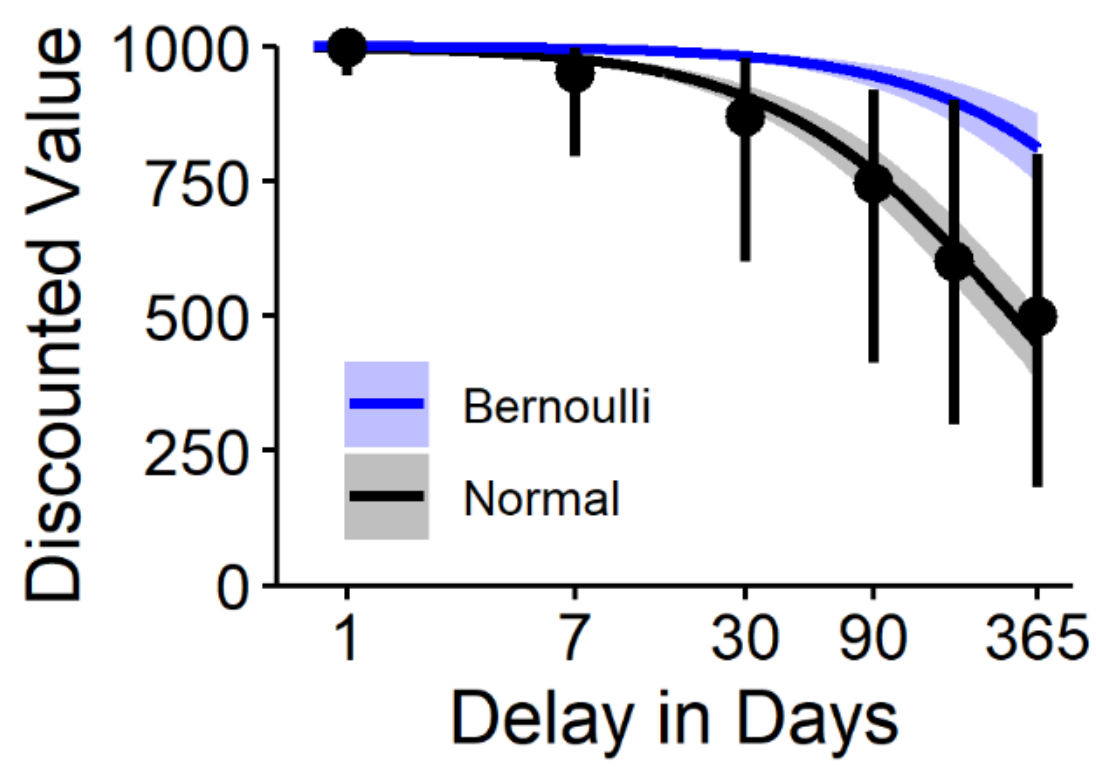

Note. Discounting curves based on k values estimated using a Bernoulli and Normal likelihood.

On the one hand, one could argue that the Bernoulli model's discounting curve is not comparable to the indifference points because the Bernoulli model is fit to the individual choice data and not the indifference points. To that point, the Bernoulli model fit the individual choice data well. On the other hand, however, indifference points on adjusting amount intertemporal choice tasks are derived from unique sequences of individual choices at each delay. That is, every possible indifference point is perfectly correlated with a unique sequence of individual choices at a given delay on the task. The discrepancy between fits from the Bernoulli

likelihood and the indifference points is likely because the Bernoulli model does not account for the sequential nature of choices on the task. In the end, however, when we fit the Bernoulli likelihood, we found the same pattern of findings as those presented in the main text: steeper discounting was associated with lower punishment learning rates ($\gamma_{A_{pun}1}$: M = -0.057, 95% HDI = [-0.081,-0.034]) and win/loss frequency sensitivities ($\gamma_{\beta f1}$: M = -0.253, 95% HDI = [-0.413,-0.091]), with a trend towards lower response biases ($\gamma_{\beta b1}$: M = -0.037, 95% HDI = [-0.075,0.006]). Furthermore, although log k values showed mean-level differences between the two estimation approaches (Bernoulli □$_{\text{log k}}$: M = -1.48, Normal □$_{\text{log k}}$: M = 0.21), they showed high rank-order consistency: r = .83, p < .001. Given these findings, we chose to present results from the model that more closely aligned with the indifference points (i.e., the Normal likelihood model).

Correlations Between Joint Model Parameters

As noted in the main text, we calculated correlations between log k values and ORL parameters to provide more intuitive measures of the relations between parameters. To do this, we calculated correlations iteration-by-iteration from the posterior distributions of the person-level parameters. This approach generates posterior distributions of correlation coefficients which can then be evaluated. Summary statistics of these posterior distributions are presented in Table 3 of the main text; however, Figure S6 shows these distributions. Overall, steep discounting (high log k) was associated with lower ORL parameters, but evidence favoring these associations was present only for punishment learning rates (A_{pun}), win/loss frequency sensitivities (□ f), and response bias (□ b).

Figure S6
Cross-Task Associations

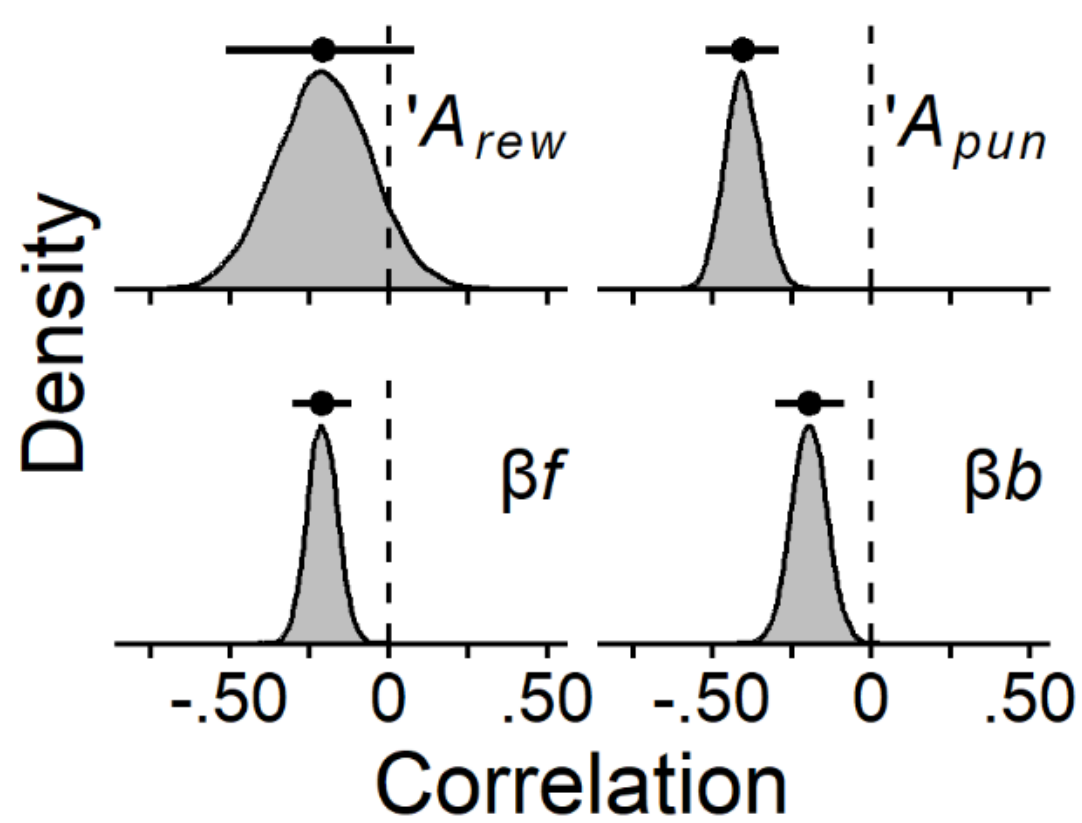

Note. Posterior distributions of the correlations between ORL parameters and log k values. Datapoint and line represent posterior mean ±95% HDIs.

Sensitivity Analyses

Model Without Outlier

As Figure 2 shows in the main text, one participant had a particularly high log k value. To ensure that our results did not depend on the presence of this participant, we reran our analyses excluding this participant. Table S5 shows summary statistics of the posterior distributions for the group-level parameters from this model, and Figure S7 shows person-level ORL parameters as a function of person-level log k values from this model. Overall, we find the same pattern of findings as displayed in the main text. Steep discounting (higher log k) was associated with elevated punishment learning rates, win/loss frequency sensitivities, and response biases, but not with reward learning rates.

Table S5

Descriptive Statistics of Posterior Distributions

Parameter	□$_0$	□$_1$	□
A_{rew}	-1.21 [-1.28,-1.14]	-0.02 [-0.06,0.01]	0.12 [0.11,0.13]
A_{pun}	-1.42 [-1.49,-1.36]	**-0.07 [-0.10,-0.04]**	0.09 [0.08,0.10]
□ f	3.43 [2.95,3.87]	**-0.31 [-0.55,-0.08]**	3.37 [2.91,3.83]
□ b	1.05 [0.94,1.16]	**-0.07 [-0.13,-0.02]**	1.03 [0.92,1.14]
Log k			0.18 [-0.08,0.43]
Log ϕ			0.58 [0.52,0.63]

Note. Group-level posterior means ±95% HDI for the model excluding the extreme participant. □s for the ORL parameters are posterior means at the group-level mean log k. □s for A_{rew} and A_{pun} are presented on the transformed scale (i.e., bounded between 0 & 1). Bold □$_1$s indicate slope effects in which the 95% HDIs did not overlap with 0, indicating evidence of an association between log k and the ORL parameter.

Figure S7
ORL Parameters as a Function of Log ks

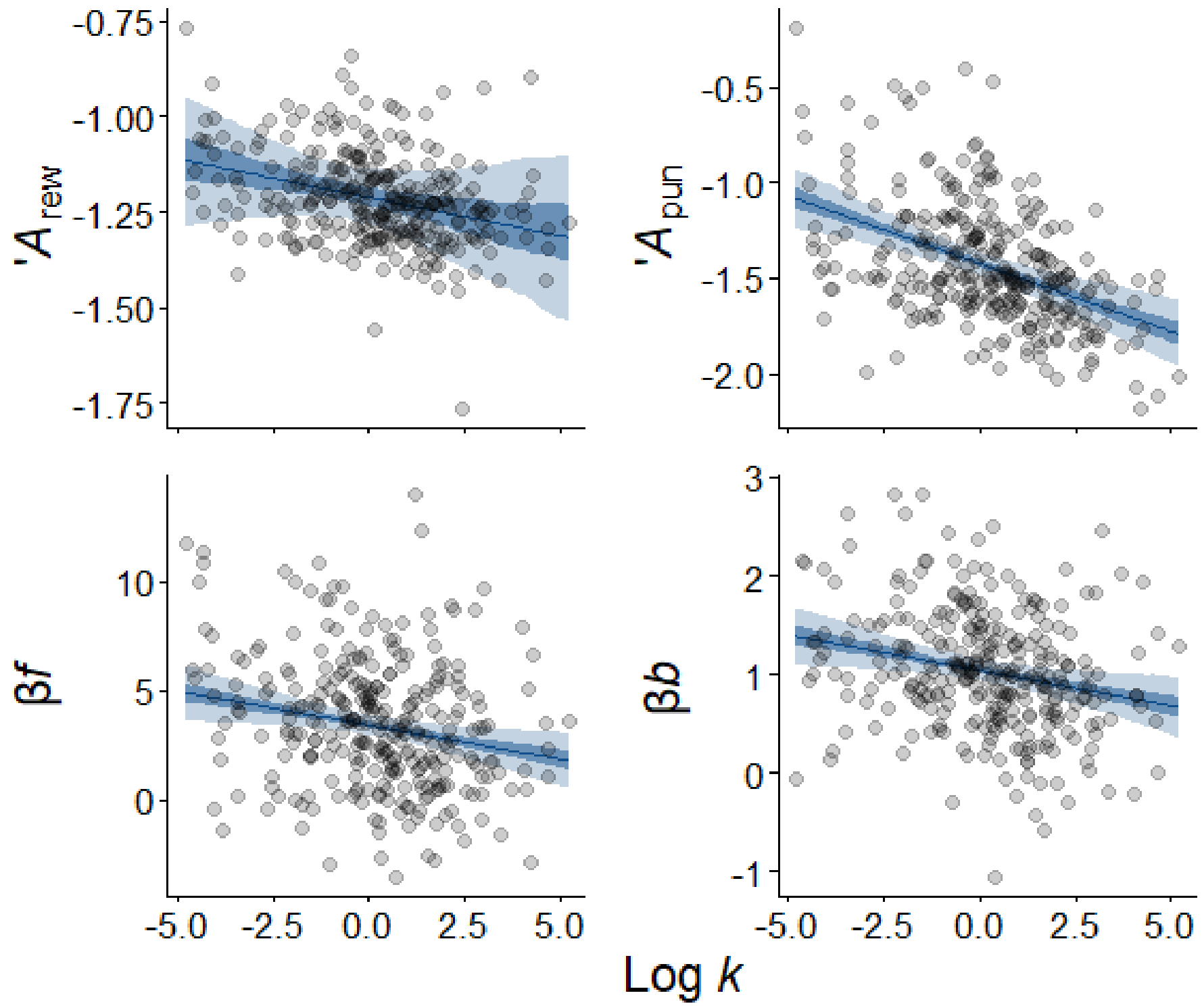

Note. Parameters from the joint model without the extreme participant. Log k values are displayed on the x-axis and ORL parameters are displayed on the y-axis. Regression lines were constructed from the □s, estimating the relation between log k and ORL parameters. Darker and lighter shaded areas represent 50% and 95% HDIs, respectively. Datapoints represent posterior means of

Non-Coupled Models

We conducted a follow-up analysis to examine whether the cross-task associations found in the joint model would replicate in non-coupled models. That is, we fit the hyperbolic discounting model to the intertemporal choice (ITC) task data and the Outcome Representation Learning (ORL) model to the Iowa Gambling Task (IGT) data separately (i.e., not in a joint model). The primary difference between the non-coupled models and the joint model was that the ORL parameters were not fit as a function of log k values from the hyperbolic discounting model. Priors for these models are shown below.

Hyperbolic Model Group-Level Priors:

$$\mu_{\log k} \sim \text{Normal}(0,1)$$
$$\mu_{\log \phi} \sim \text{Normal}(0,0.5)$$
$$\sigma_{\log k}, \sigma_{\log \phi} \sim \text{Half-Normal}(0.5)$$

Hyperbolic Model Person-Level Priors:

$$\log k_i \sim \text{Normal}(\mu_{\log k}, \sigma_{\log k})$$
$$\log \phi_i \sim \text{Normal}(\mu_{\log \phi}, \sigma_{\log \phi})$$

ORL Model Group-Level Priors:

$$\mu'_{A_{rew}}, \mu'_{A_{pun}}, \mu_{\beta f}, \mu_{\beta b} \sim \text{Normal}(0,1)$$
$$\sigma'_{A_{rew}}\ \sigma'_{A_{pun}} \sim \text{Half-Normal}(0.2)$$
$$\sigma_{\beta f}, \sigma_{\beta b} \sim \text{Half-Cauchy}(1)$$

ORL Model Person-Level Priors:

$$\Phi^{-1}(A_{rew}) \sim \text{Normal}(\mu'_{A_{rew}}, \sigma'_{A_{rew}})$$
$$\Phi^{-1}(A_{pun}) \sim \text{Normal}(\mu'_{A_{pun}}, \sigma'_{A_{pun}})$$
$$\beta f \sim \text{Normal}(\mu_{\beta f}, \sigma_{\beta f})$$
$$\beta b \sim \text{Normal}(\mu_{\beta b}, \sigma_{\beta b})$$

After fitting the models, we correlated log k values with ORL parameters. To do this, we first calculated person-level point estimates for each parameter by computing posteriors means for the parameters. Next, we correlated the log k point estimates with point estimates for each ORL parameter.

Overall, we replicated the main findings from the joint model. As shown in Figure S8, steep discounting (higher log k) was significantly correlated with lower punishment learning rates ($r = -.24$, $p < .001$) and lower win-/loss-frequency sensitivities ($r = -.20$, $p < .001$); however, steep discounting was not correlated with reward learning rates ($r = -.01$, $p = .81$) nor response biases ($r = -.11$, $p = .06$). As the figure illustrates, there is no association between reward learning rates (A_{rew}) and log k values. Although there is a trend in which steeper discounting is associated with lower response biases (□ b), similar to the joint model, this correlation was weak and not statistically significant. In sum, we replicated our main findings from the joint model using non-coupled models in which ORL parameters were not estimated as a function of the log k values.

Figure S8
ORL Parameters as a Function of Log ks for Non-Coupled

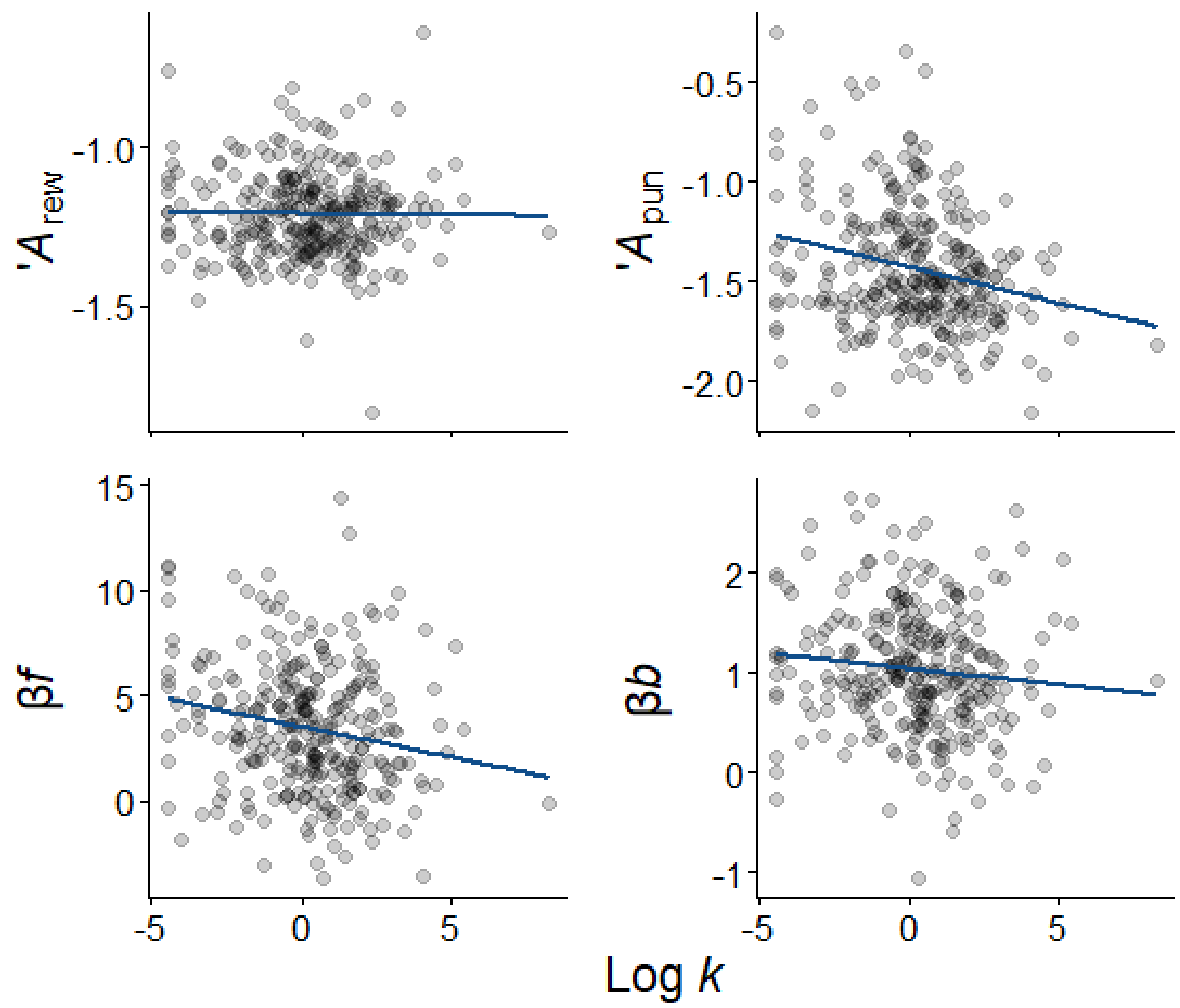

Note. Parameters from the non-coupled model with log k values on the x-axis and ORL parameters on the y-axis. Datapoints represent posterior means of individual participant parameters and regression

No-Dyad Model

As described in the main text, some participants were part of mother-father dyads (N = 68 dyads) and thus data from these participants may not be independent. We initially assessed correlations between parameters from participants within dyads, shown in Table S6. Only log k values were significantly correlated between members of the dyads. In addition, all correlations were in the low-to-moderate range and may not be problematic in terms of collinearity (Dormann et al., 2013).

Table S6
Correlations Between Dyad Parameters

Parameter	r	p
A_{rew}	-.001	.993
A_{pun}	.215	.079
□ f	.176	.152
□ b	.126	.306
Log k	**.358**	**.003**
Log ϕ	-.085	.489

Note. Correlations between parameters from mothers and fathers within a dyad.

Parameter values were calculated as posterior means and then correlated.

To be conservative, we conducted a follow-up analysis in which we randomly removed a single participant from each dyad and refit the joint model. Results of this model show that the associations between discounting and ORL model parameters are weakened compared to our main findings from the text. In general, however, we replicate the finding that steep discounting (i.e., higher log k values) is associated with lower punishment learning rates (A_{pun}), shown in Table S7. In addition, we constructed regression plots, depicted in Figure S9, illustrating the associations between log k values and ORL parameters. Visually, these plots show that results from this model are generally consistent with those presented in the main text, particularly with respect to the association between punishment learning rates and delay discounting.

Table S7
Descriptive Statistics of Posterior Distributions

Parameter	□$_0$	□$_1$	□
A_{rew}	-1.25 [-1.34,-1.17]	-0.03 [-0.07,0.01]	0.11 [0.09,0.13]
A_{pun}	-1.50 [-1.57,-1.43]	**-0.05 [-0.08,-0.02]**	0.07 [0.07,0.08]
□ f	3.18 [2.69,3.68]	-0.23 [-0.48,0.02]	3.11 [2.61,3.62]
□ b	0.92 [0.81,1.04]	-0.02 [-0.08,0.03]	0.92 [0.80,1.03]
Log k			0.27 [-0.03,0.55]
Log ϕ			0.59 [0.52,0.65]

Note. Group-level posterior means ±95% HDI for the model excluding one participant from each dyad. □s for the ORL parameters are posterior means at the group-level mean log k. □s for A_{rew} and A_{pun} are presented on the transformed scale (i.e., bounded between 0 & 1). Bold □$_1$s indicate slope effects in which the 95% HDIs did not overlap with 0, indicating evidence of an association between log k and the ORL parameter.

Figure S9
ORL Parameters as a Function of Log ks

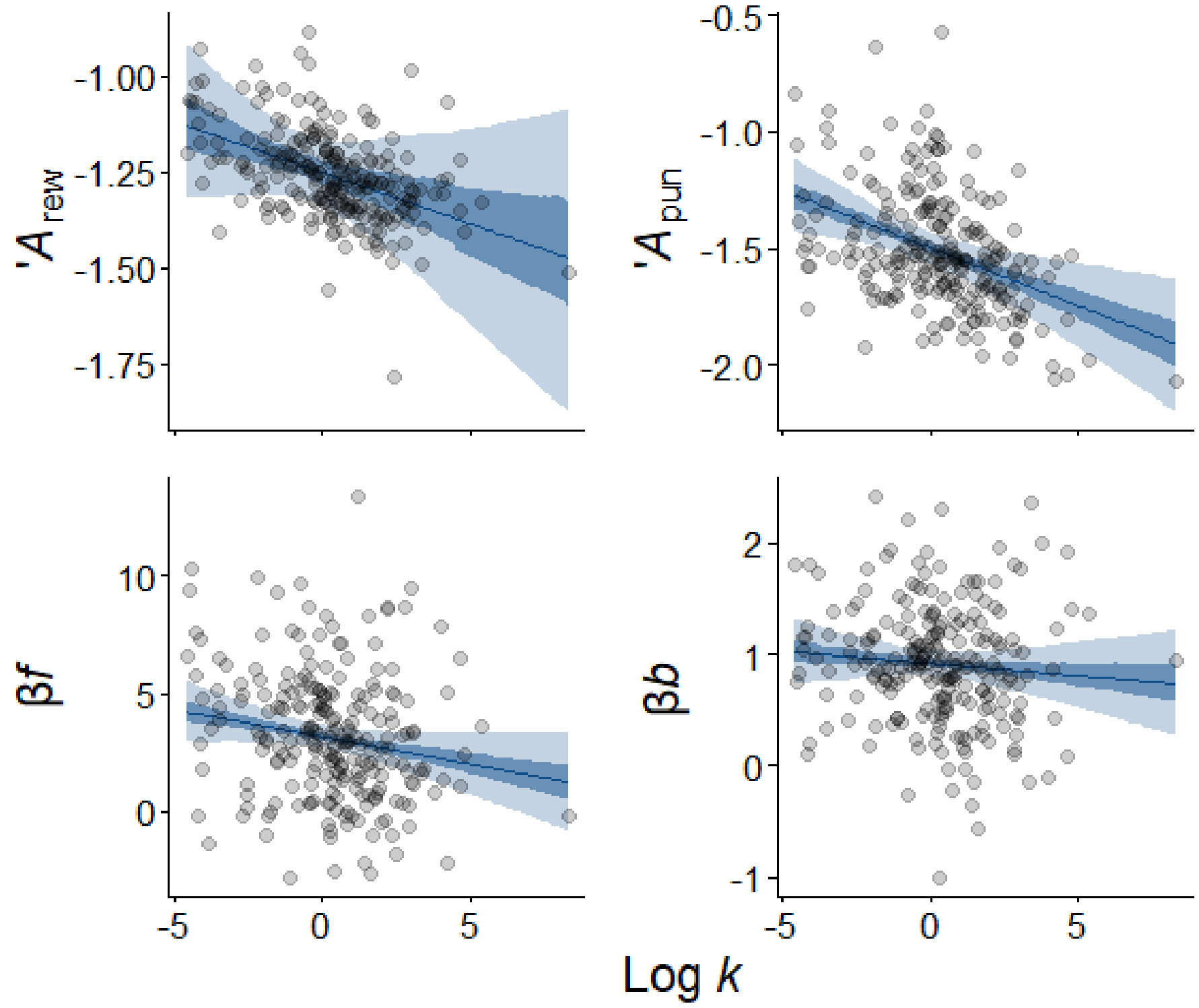

Note. Parameters from the joint model excluding one participant from each dyad. Log k values are displayed on the x-axis and ORL parameters on the y-axis. Regression lines were constructed from the □s, estimating the relation between log k and ORL parameters. Darker and lighter shaded areas represent 50% and 95% HDIs, respectively. Datapoints represent posterior means of individual participant
