# Supplementary material for: Associations Between Steep Delay Discounting and Punishment Learning
Source: Comput Psychiatr. 2026 Sep 1;10(1):159–83. doi: 10.5334/cpsy.172 (PMC13544050; doi:10.5334/cpsy.172)
Supplement: Peer review history

# Reviewer report: Round 1, Reviewer C

**DOI:** 10.5334/cpsy.172.r1

## Recommendation:

Accept with Major Revision

## Comments/Explanation (required). Please include references to any minor or major revisions required, as well as addressing any issues with language clarity. Authors will see these comments:

The manuscript entitled “Associations Between Steep Delay Discounting and Punishment Learning ” examines delay discounting and reinforcement learning in a common study and suggests that steeper delay discounting and weaker learning from punishment are associated. The study combines an intertemporal choice task and the Iowa Gambling Task. It relies on a hierarchical and joint modeling approach.

Overall, I found the manuscript to be clearly written, and the authors use state-of-the-art computational modeling methods. I have comments on the interpretation of the results, the statistical analyses, and model validation.

1. Behavioral evidence of a relationship between delay discounting and punishment learning

The conclusion that delay discounting and punishment learning are associated with each other is primarily based on the computational modeling results. However, in my opinion, especially when considering real-world and clinical implications, computational differences need to translate into observable differences in choice behavior. Figure 4 goes in this direction, showing choice behavior of example participants with shallow and steep discounting. However, the corresponding section on “Individual Participant Data” (page 17) does not provide any formal statistical analysis of group-level data. I would recommend a systematic analysis of the behavioral links between the two constructs across participants. I think it would be especially important to better understand if higher delay discounting is associated with higher or lower IGT performance.

Also, the results on page 16 describing Figure 1 are not quantified. I think basic performance analyses should be backed up by inferential statistics.

2. Interpretation of the learning rates

Reinforcement learning tasks, including the IGT, are often treated as if they all measure the same underlying construct. However, mounting evidence suggests that learning rates are highly task-dependent and that generalization across tasks is surprisingly weak (Eckstein et al., 2021). To better understand which learning rate magnitude is beneficial in the present task, it would be very useful to see a simulation study demonstrating the link between learning-rate magnitude and choice performance.

3. Split-half reliability

A related point is that the reliability of reinforcement learning tasks is often quite low. I have seen a citation of Haynes et al. (2024) on test-retest reliability with likely the same task. Can the authors please clarify whether they have systematically evaluated the test-retest or split-half reliability of the present task?

4. Parameter recovery

Page 11: The authors write that they show parameter recovery analyses. However, I could not find them in the supplement. I hope I didn’t miss anything, but I think it would be important to ensure that recovery analyses are included.

5. Play-or-pass outcome representation learning

The learning model (shown in Table 2) combines the expected value (computed with a standard RL model with two learning rates) and expected win/loss frequencies for chosen and non-chosen decks (yielding the “value” variable). The motivation for this particular model, and why the authors don’t just use a standard RL model without frequencies, remains partly elusive (though in the Introduction, the authors explain that participants seem to favor more frequently rewarded decks independent of magnitude). It would certainly help to explain the model in more detail in the methods rather than just showing equations in Table 2. Moreover, it would be useful to run a model comparison between the ORL model and a standard RL model with one learning rate, and an RL model with separate positive and negative learning rates.

6. Does the correlation between ORL parameters and log k values replicate in a non-coupled model?

I see the benefits of joint modeling for the present study. But complex, hierarchical models can sometimes work in less predictable ways than simpler models. I would suggest a control analysis without a joint model to see if correlations replicate without direct coupling during parameter estimation. This would demonstrate whether the finding is directly conditional on joint modeling or holds across approaches.

References

Eckstein, M. K., Wilbrecht, L., & Collins, A. G. (2021). What do reinforcement learning models measure? Interpreting model parameters in cognition and neuroscience. Current Opinion in Behavioral Sciences, 41, 128-137.

# Reviewer report: Round 1, Reviewer G

**DOI:** 10.5334/cpsy.172.r2

## Recommendation:

Accept with Major Revision

## Comments/Explanation (required). Please include references to any minor or major revisions required, as well as addressing any issues with language clarity. Authors will see these comments:

In this study, the authors investigate whether and how computational metrics characterizing delay discounting and learning/decision-making on an adapted version of the Iowa Gambling Task relate to one another in a large convenience sample composed of parents of children involved in another study (N=299, 26% male). Using clever modelling techniques, they show that steeper delay discounting (high log k) is associated with lower punishment learning rates, sensitivity to win and loss frequency, and response biases as estimated from the IGT. Overall, the manuscript is well written, straightforward, and methodologically sound, with limitations clearly acknowledged. I have some concerns/questions regarding the conceptual rationale, some modelling choices, and a few interpretations, as well as minor comments on precision of the prose / concreteness of the arguments. If these are addressed, I see no major hurdles to publication unless editors prioritise novelty/ clear implications for psychiatry.

Introduction:

- P.2.: The argument is a little bit thin and could use fleshing out. What is the theory linking learning and delay discounting?
- P.2.: Likewise, the prose is somewhat imprecise. Who’s “sharing goals” with whom and why does it matter? What are the “focal aspects of behaviour” the authors mention? “More refined” than what?
- P.3.: Be more explicit: what is the supposed reason that steep DD might contribute to mental health issues? Is it that steep DD might lead to impulsive/risky decisions (how?) and/or that the consequences of these decisions lead to mental health issues?
- P.3.: “participants make a series of choices between uncertain outcomes” – people choose between options, not outcomes, especially if there is no deterministic relationship between option and outcome
- P.3.: please clarify what the differential contributions are of the ventromedial and ventrolateral PFC to IGT performance and DD.
- P.5/6.: please motivate why the computational approach will help make sense of mixed results in previous studies. Can you speculate what these studies might have missed that the authors are able to see using the computational approach? E.g., “traditional scoring measures (e.g., number of choices for good decks) cannot differentiate approaching the good decks from avoidance of the bad decks” ◊ “if participants did [approach hypothesis] and [avoidance hypothesis], this would come out as a null result in traditional analyses.” (or similar); same for the win/loss sensitivity, response bias…
- P.7.: “More generally, computational model parameters provide more reliable and predictively valid measures of critical cognitive processes” ◊ this is overly general. Which exact computational models and parameters (!) are we talking about? The term is very broad and can encompass everything from a fancy regression to sophisticated Bayesian inference accounts with multiple levels of uncertainty, which surely differ in their reliability and predictive validity? Also perhaps depending on the experimental task they’re applied to?
- P.8.: “Thus, these studies show the utility of joint modeling for not only assessing test-retest reliability, but also for assessing convergence (or divergence) across psychological constructs.” I buy the argument but as mentioned above I’m missing some more specific arguments as to how the authors think reward valuation and learning from rewards and punishments might be related and why, and what might be aspects that differentiate between them. What are the authors’ hypotheses?

Methods

- P.8.: The authors state that data from the play-or-pass IGT have been presented elsewhere. Can they clarify whether this means that they are republishing data? If so, can they clarify where the data were published? Also, do the authors see any benefit in waiting for the longitudinal data to come in and publishing it together rather than bit by bit?
- P.9.: Can the authors clarify whether there were in- and exclusion criteria for the parents’ children only or whether there were specific in- and exclusion criteria for the parents as well? And please indicate whether and how the authors think these criteria affect their adult sample, can these be understood as “healthy adults”? Most importantly, can the authors clarify whether for some families, both mother and father were included in the study and whether this was accounted for in the analyses (as these are then no longer independent from one another).
- P.9.: The authors mention self-report questionnaires, but they never seem to crop up. Given that they investigate DD and learning as hypothesized etiological factors for mental health issues, could some of these questionnaires be relevant here? What were they?
- P.10.: Can the authors state how many trials there were in the IGT?
- P.11.: Why do the authors show the parameters in centered rather than native space?
- P.11.: I think the reasoning behind the choice of priors should go in the main manuscript.
- P.11.: Please report correlations between simulated and observed data and show scatter plots.
- P.15.: “To further contextualize the between-task associations, we calculated follow-up correlations between discounting parameters and ORL parameters, and between discounting parameters and behavioral measures of IGT performance.” Please comment on whether/to what extent that constitutes double dipping if the slope is already calculated as part of the model. Wouldn’t the (standardized) average slope be the same as the correlation?
- P.16.: Since the ORL is rather central in the paper, it would be good to have a more intuitive explanation as to what it does in addition to the maths in table 2. Can the authors also comment on whether they considered other models for the DD and IGT tasks and, if not, why not? E.g., are the response bias / win loss frequency sensitivity / differential LRs for wins/losses really necessary; is there a choice stochasticity/sensitivity term? If not, why not etc.

Results

- P.16.: “Overall, steep delay discounting (i.e., a higher log k) was associated with lower reward and punishment learning rates (Arew & Apun), win/loss frequency sensitivities (βf), and response biases (βb).” – have the authors checked whether this association is at all related to model fit? As in, might participants with higher log k just behave more randomly on the IGT?

Discussion

- P.19. My understanding is that RL parameters tend to generalize poorly across tasks (with the exception of the inverse temperature/ decision noise/ choice stochasticity/ choice sensitivity parameters). Can the authors comment on what this means with regard to inferences from this specific study employing a specific version of the IGT to learning in general as well as the PVS and NVS globally?
- P.19.: Vague: “Overall, our findings suggest that learning on the IGT and reward valuation on ITC tasks contribute to overlapping patterns of behavior, advancing our understanding of the RDoC PVS and NVS” – as mentioned above wrt the introduction, I’m missing a bit of theory here. How do they relate and how does that make sense conceptually?
- P.19.: “our findings may be helpful in understanding how dysfunctions in these processes may contribute to mental illness” – callback to my point about questionnaires from earlier. Also, can the authors specify in what way their findings will be helpful? Not trying to be facetious, it would just be good to understand how a more complete picture of the interrelationships between DD and learning may help understand dysfunctions underlying psychiatric symptoms.
- P.20.: “Our data supports this perspective, such that people who showed steeper discounting were less sensitive to punishment on the IGT in terms of loss magnitude (lower Apun) and loss frequency (lower βf).” … but also lower sensitivity to win frequency. So perhaps generally lower sensitivity to feedback? / more randomness?
- P.20. Have the authors considered looking at delay discounting for losses as well as wins? Would this not more directly test their hypothesis that “populations of people who show steep discounting […] may be insensitive to the losses occurring over time that ultimately lead to larger negative consequences experienced later”?
- P.21.: “dysfunctional reward and punishment learning are independently associated with psychopathology (e.g., substance abuse)” – is it true that they are clearly *independently* associated with psychopathology or just in separate studies? Either way this needs a reference.
- In general, perhaps the authors might want to comment on whether delay discounting should always be considered irrational/impulsive. There might be environments where DD is in fact very rational – e.g., a high inflation environment, if the agent thinks they can invest the money more profitably than the game does, if the environment is unreliable (e.g., the money may not be paid out), if the agent’s horizon is diminished (due to terminal illness, living in a warzone etc.)… This might be good to clarify but also provide a handle on why some people might develop steep DD in the first place.

# Reviewer report: Round 2, Reviewer A

**DOI:** 10.5334/cpsy.172.r3

## Recommendation:

Accept with Minor Revision

## Comments/Explanation (required). Please include references to any minor or major revisions required, as well as addressing any issues with language clarity. Authors will see these comments:

This is the second review of the manuscript on “Associations Between Steep Delay Discounting and Punishment Learning”. I thank the authors for their careful and comprehensive revisions. Except for one minor point, they have addressed all my previous concerns.

The only remaining point is that I recommend that the authors present statistical analyses of the empirical play proportion data, in particular, whether the different decks differ in their play probabilities (Figure 1). In response to my earlier comment on this (comment #ii), the authors added “posterior p-values” for the posterior predictive checks. However, my point was purely about the empirical data, where, so far, the play proportions are not statistically compared between the decks.

# Reviewer report: Round 2, Reviewer B

**DOI:** 10.5334/cpsy.172.r4

## Recommendation:

Accept with Minor Revision

## Comments/Explanation (required). Please include references to any minor or major revisions required, as well as addressing any issues with language clarity. Authors will see these comments:

I thank the authors for their thorough revision of their manuscript. They adressed most of my points satisfactorily. I have a few more (minor) comments (attached) but would generally reccomend publication, assuming the editors are happy to publish this part of the data on its own and do not insist that the full longitudinal data be published in a single manuscript.

# Reviewer report: Round 1, Author Response

**DOI:** 10.5334/cpsy.172.r5

We would first like to thank the reviewers for their comments aimed at strengthening our manuscript. Given the specific feedback on prose, clarifying the goals of the manuscript, and feedback on the analyses, we have made substantial revisions to the Introduction, added additional sections to the Method, Results, and supplement, as well as revised portions of the Discussion. Below, we list reviewer comments (bolded) along with our responses (unbolded). In addition, we provide excerpts from the manuscript to illustrate some of the changes (blue font).

**Reviewer 1**

## Reviewer C:

1A) Behavioral evidence of a relationship between delay discounting and punishment learning.

i. The conclusion that delay discounting and punishment learning are associated with each other is primarily based on the computational modeling results. However, in my opinion, especially when considering real-world and clinical implications, computational differences need to translate into observable differences in choice behavior. Figure 4 goes in this direction, showing choice behavior of example participants with shallow and steep discounting. However, the corresponding section on “Individual Participant Data” (page 17) does not provide any formal statistical analysis of group-level data. I would recommend a systematic analysis of the behavioral links between the two constructs across participants. I think it would be especially important to better understand if higher delay discounting is associated with higher or lower IGT performance.

## Author’s response

Thank you for discussing the importance of aligning computational differences with behavioral differences. We wholeheartedly agree and have added further detail to the analysis of behavior (pp. 23-25), including correlations with summary measures of behavior as well as a Figure (Figure 4) displaying associations between summary measures of IGT performance and discounting. To briefly summarize, people who show steep discounting earned less in the IGT, *r* = –.18, *p* < .01. In addition, we found that steep discounting was associated with playing less often on the good decks, *r_S_* = –.21, *p* < .001, but was not associated with playing on the bad decks, *r_S_* = .10, *p* = .08. This latter finding is counterintuitive given our computational results; that is, lower punishment learning rates associated with steep discounting, but no association with reward learning rates. To probe this further, we calculated differences scores for each participant by computing the difference in playing from block 1 of the IGT to block 3 of the IGT for the good and bad decks. Although weak, difference scores for the bad decks were significantly correlated with delay discounting, *r_S_* = .21, *p* < .01, such that more shallow discounting (lower log k) was associated with a greater reduction in playing on the bad decks from the first to the last block of trials which aligns with the computational results.

## Reviewer C:

ii. Also, the results on page 16 describing Figure 1 are not quantified. I think basic performance analyses should be backed up by inferential statistics.

## Author’s response

To provide a quantitative measure of how well posterior predictions align with observed data, we calculated posterior “*p*-values” (Gelman et al., 2014). In brief, we computed the proportion of predicted posterior predictions below the observed data. Models that predict the data well should have *p*-values closer to .5 whereas models that do not predict the data would have *p*-values closer to 0, indicating posterior predictions underestimate the observed data, or 1, indicating posterior predictions overestimate the observed data. Posterior *p*-values for the IGT and ITC task data were .470 and .498, respectively, indicating that posterior predictions aligned with the observed data. Further details of the posterior predictive checks are described in the Method (pp. 19-20) and the Results (p. 22).

## Reviewer C:

1B) Interpretation of the learning rates.

Reinforcement learning tasks, including the IGT, are often treated as if they all measure the same underlying construct. However, mounting evidence suggests that learning rates are highly task-dependent and that generalization across tasks is surprisingly weak (Eckstein et al., 2021). To better understand which learning rate magnitude is beneficial in the present task, it would be very useful to see a simulation study demonstrating the link between learning-rate magnitude and choice performance.

## Author’s response

This is a great point. Per the reviewer’s suggestion we have added a simulation study to the supplement (pp. 1-2) and reference the simulation in-text where relevant. In brief, the simulation study shows that elevated punishment learning rates and win/loss frequency sensitivities are associated with a more sharp decline in playing on the bad decks (see Figure S1).

## Reviewer C:

1C) Split-half reliability.

A related point is that the reliability of reinforcement learning tasks is often quite low. I have seen a citation of Haynes et al. (2024) on test-retest reliability with likely the same task. Can the authors please clarify whether they have systematically evaluated the test-retest or split-half reliability of the present task?

## Author’s response

This point is also related to some of Reviewer 2’s concerns (see points 2A.vii. & 2D.i. below). These are valid concerns because, as noted, RL parameters notoriously have weak test-retest reliability (Karvelis et al., 2023) and poor generalizability (Eckstein et al., 2021). We point out these issues as part of the limitations (pp. 34-35) and discuss reliability of measures from both tasks. To specifically address the concern of ORL parameter reliability, we provide more detail of Haynes et al. (2024) who assessed test-retest reliability of both traditional scoring measures and ORL parameters from the same play-or-pass IGT in a sample of college students. Thus, the task was the same, but those data came from a different sample. Generally, they found that play-or-pass IGT measures were reliable, but ORL parameter reliability drops substantially when parameters are estimated without a joint probabilistic structure defining parameters across two sessions. Reliability should improve with cross-task joint modeling, as in the present study (Haines et al., 2025); however, we are unaware of studies directly illustrating that cross-task joint modeling empirically improves reliability. Given these concerns, we urge caution in generalizing beyond our study.

pp. 34-35: [M]easures from the Iowa Gambling Task (IGT) are mixed in terms of rank-order consistency across time (Buelow & Barnhart, 2018; Schmitz et al., 2020) and reinforcement learning (RL) parameters are known to have poor reliability and generalizability (Eckstein et al., 2022; Karvelis et al., 2023), suggesting that our findings may not generalize beyond the present study, particularly with different IGT-like tasks. It is worth noting that Haynes et al. (2024) assessed test-retest reliability of measures from the same play-or-pass IGT used in the present study in college students, with some promising findings (*r*s ≥ .49 for summary measures, *r*s ≥ .59 for ORL parameters). However, although significant, they found that rank-order stability degrades substantially when ORL parameters are estimated without a joint probabilistic structure defining parameters across two sessions (*r*-values = .21-.35; see their supplement). Joint modeling across tasks, as in this study, should improve parameter reliability (Haines et al., 2025), but we urge caution when generalization beyond this study, particularly with respect to measuring RDoC constructs in the Positive and Negative Valence Systems (e.g., punishment learning), as differences across time or tasks (e.g., different RL tasks) may not replicate.

## Reviewer C:

1D) Parameter recovery.

Page 11: The authors write that they show parameter recovery analyses. However, I could not find them in the supplement. I hope I didn’t miss anything, but I think it would be important to ensure that recovery analyses are included.

## Author’s response

Thank you for pointing this out; they were inadvertently omitted from the initial submission. Parameter recovery diagnostics have now been included in the supplement (p. 3). In brief, parameter recovery was strong for most parameters (*r*s between recovered & true parameters ≥ .65), although reward learning rates tended to show the poorest parameter recovery: *r* = .47 for correlation between “true” and “recovered” reward learning rates.

## Reviewer C:

1E) Play-or-pass outcome representation learning

The learning model (shown in Table 2) combines the expected value (computed with a standard RL model with two learning rates) and expected win/loss frequencies for chosen and non-chosen decks (yielding the “value” variable). The motivation for this particular model, and why the authors don’t just use a standard RL model without frequencies, remains partly elusive (though in the Introduction, the authors explain that participants seem to favor more frequently rewarded decks independent of magnitude). It would certainly help to explain the model in more detail in the methods rather than just showing equations in Table 2. Moreover, it would be useful to run a model comparison between the ORL model and a standard RL model with one learning rate, and an RL model with separate positive and negative learning rates.

## Author’s response

In line with Reviewer 2’s comments regarding the use of the ORL model, we provide further detail on why we chose this model in the Introduction, with a particular focus on separating learning from gains and losses as well as accounting for win/loss frequency sensitivity. To do this, we discuss the shortcomings of traditional scoring measures (excerpt below; pp. 6-7) as well as how the ORL is designed to address these issues (pp. 8-9):

pp. 6-7: [P]erformance on the IGT is typically aggregated into summary measures of behavior (e.g., net score across the whole session) describing how well participants did on the task, but the IGT is a complex task thought to rely on multiple behavioral processes that are not captured by these summary measures (Ahn et al., 2016; Mukherjee & Kable, 2014). For example, each deck within the IGT consists of both gains and losses which vary in magnitude and frequency; however, gains and losses influence behavior asymmetrically (Fontes & Shahan, 2021; Kahneman & Tversky, 1979) and sensitivity to win/loss frequency, irrespective of the magnitude of those wins and losses, has a strong effect on decision-making in the IGT (Chiu et al., 2008; Chiu & Lin, 2007; Steingroever et al., 2013; Wetzels et al., 2010).

In addition, we explain the ORL model in more detail in the Method (pp. 15-17) and, per the reviewer’s suggestion, we conducted model comparisons using leave-one-out-information-criteria (LOOIC) between the ORL and the two simpler RL models. In one model (labeled the 2-parameter RL model), we estimated a single learning rate and a bias parameter from the IGT data, and in the other model (labeled the 3-parameter RL model), we estimated a reward learning rate, punishment learning rate, and bias parameter. When fitting without the bias parameter, the models did not perform well and we did not believe this served as a proper test to determine whether, for example, the win/loss frequency sensitivity parameter was justified. Results of these model comparisons are presented in the supplement (pp. 3-5). To summarize, the ORL model outperformed both of these simpler RL models. LOOICs are interpreted similar to deviances and the ORL had a lower LOOIC than the simpler RL models by a difference > 1940. Importantly, the 3-parameter model is nearly identical to the ORL, except win/loss frequency sensitivity is not included. Therefore, our finding that the ORL outperforms the 3-parameter RL model suggests that including win/loss frequency sensitivity is warranted.

## Reviewer C:

1F) Does the correlation between ORL parameters and log k values replicate in a non-coupled model?

I see the benefits of joint modeling for the present study. But complex, hierarchical models can sometimes work in less predictable ways than simpler models. I would suggest a control analysis without a joint model to see if correlations replicate without direct coupling during parameter estimation. This would demonstrate whether the finding is directly conditional on joint modeling or holds across approaches.

## Author’s response

Thank you for this suggestion. We conducted the analysis using the non-coupled approach and found replication across approaches: steeper discounting was associated with lower punishment learning rates and win frequency sensitivities, with a trend towards lower response biases. As with the joint model, reward learning rates were not associated with log k values. We have noted this in the main text as part of the Sensitivity Analyses (pp. 25-26), with further details in the supplement (pp. 12-14). For comparison, the table below correlations from the joint and non-coupled models side-by-side. Joint model correlations are posterior means with 95% HDIs. Non-coupled model correlations are Pearson correlation coefficients with 95% confidence intervals. Bolded values represent “significant” correlations.

**Table:** 


ORL Parameter	Model	

	Joint Model	Non-Coupled Models

*A_rew_*	–.20 [–.48,.10]	–.01 [–.13,.10]
*A_rew_*	**–.40 [–.52,–.29]**	**–.24 [–.35,–.13]**
*βf*	**–.21 [–.30,–.12]**	**–.20 [–.31,–.08]**
*βb*	**–.20 [–.30,–.09]**	–.11 [–.22,.01]


## Reviewer G:

**Reviewer 2**

2A) Introduction: We appreciate Reviewer 2’s comments on improving the prose and directing us to specific places that could be strengthened. We have substantially restructured the Introduction in response to these comments. Revisions addressing some of these comments are more integrated throughout the Introduction. Thus, we point out specific excerpts relevant to this Reviewer’s comments but would like to note that the Introduction as a whole has been restructured to address these comments.

i. P.2.: The argument is a little bit thin and could use fleshing out. What is the theory linking learning and delay discounting?

## Author’s response

We added additional text describing the logic of prior research assessing the association between delay discounting and learning on the Iowa Gambling. This has been integrated in various sections of the Introduction, but to briefly summarize, delay discounting may be implicated in learning on the IGT such that steep discounting may explain why people would prioritize short-term gains at the cost of long-term negative outcomes in the IGT.

Example excerpt on pp. 4-5: Because the decks vary in their short- and long-term gains and losses, performance on the IGT has been described as a measure of how sensitive people are to consequences that accumulate over time (Bechara et al., 1994; Chiu et al., 2008). Assuming that better IGT performance relies on sensitivity to delayed outcomes, we would expect steep discounting (i.e., preference for immediacy) to be associated with failing to learn which decks are good and bad on the IGT.

…

[S]tudies suggest that learning on the IGT is related to sensitivity to delayed consequences, as measured by delay discounting; however, some studies show little-to-no relation between the degree of discounting and IGT performance (Dom et al., 2007; Lamm et al., 2006), suggesting further research may be necessary to characterize these associations.

## Reviewer G:

ii. P.2.: Likewise, the prose is somewhat imprecise. Who’s “sharing goals” with whom and why does it matter? What are the “focal aspects of behaviour” the authors mention? “More refined” than what?

## Author’s response

In this introductory paragraph, we highlight that prior research suggests that IGT performance is related to delay discounting but that summary measures of learning cannot characterize what specific aspects of learning relate to delay discounting. This is particularly important for the IGT as performance on the task reflects multiple processes (e.g., win/loss frequency sensitivity). We clarify that the goal of this manuscript is to identify the specific learning processes on the IGT that are related to discounting:

p. 2: Computational methods allow us to break down IGT performance into more fine-grained patterns of behavior which we expected may be differentially associated with delay discounting. Because measures of delay discounting and IGT performance are widely associated with psychopathology, discussed further below, identifying how delay discounting relates to specific aspects of learning on the IGT may have implications for understanding unique patterns of suboptimal behavior associated with mental illness.

The ultimate goal of doing so is to understand how specifying these associations can clarify our understanding of the role of discounting in mental illness:

p. 4: One approach to clarifying how delay discounting may be involved in psychopathology involves using multitask assessments to clarify how discounting relates to other presumably overlapping psychological constructs (Bailey et al., 2021).

## Reviewer G:

iii. P.3.: Be more explicit: what is the supposed reason that steep DD might contribute to mental health issues? Is it that steep DD might lead to impulsive/risky decisions (how?) and/or that the consequences of these decisions lead to mental health issues?

## Author’s response

We added additional explanation regarding perspectives on how delay discounting relates to mental health problems. In general, we have described this as delay discounting being a measure of impulsive behavior that predisposes individuals to externalizing disorders, namely substance abuse, whereas, for internalizing disorders, delay discounting may be better considered as a “marker” for dysfunctional reward-related processes. See pp. 3-4.

## Reviewer G:

iv. P.3.: “participants make a series of choices between uncertain outcomes” - people choose between options, not outcomes, especially if there is no deterministic relationship between option and outcome

## Author’s response

Thank you for pointing out this area to be more precise. Through revising this section, we deleted this text.

## Reviewer G:

v. P.3.: please clarify what the differential contributions are of the ventromedial and ventrolateral PFC to IGT performance and DD.

## Author’s response

We refocused this section to discuss the correspondence of activity in the VMPFC during intertemporal choice tasks and the Iowa Gambling Task.

P. 6: Second, brain mechanisms involved in delay discounting overlap with those involved in the IGT, particularly the ventromedial prefrontal cortex (vmPFC). The vmPFC is thought to encode the subjective value of rewards (Hare et al., 2009; Kable & Glimcher, 2007), and dysfunction in the vmPFC is associated with both steeper discounting (Ciaramelli et al., 2021; Mok et al., 2021) and worse performance on the IGT (Bechara et al., 1994; Fellows & Farah, 2005; Mukherjee & Kable, 2014).

## Reviewer G:

vi. P.5/6.: please motivate why the computational approach will help make sense of mixed results in previous studies. Can you speculate what these studies might have missed that the authors are able to see using the computational approach? E.g., “traditional scoring measures (e.g., number of choices for good decks) cannot differentiate approaching the good decks from avoidance of the bad decks” ◊ “if participants did [approach hypothesis] and [avoidance hypothesis], this would come out as a null result in traditional analyses.” (or similar); same for the win/loss sensitivity, response bias…

## Author’s response

Through reframing, as suggested above, we describe in more detail the complexity of the IGT and that the task is typically thought to rely on multiple behavioral processes (e.g., reward learning, loss learning, win/loss frequency sensitivity) as well as how failing to account for this may be problematic (pp. 6-7).

Excerpt from p. 7: Failing to account for the asymmetry between learning from gains and losses and their frequencies could explain why some prior research has found no association between learning on the IGT and delay discounting (Dom et al., 2007; Lamm et al., 2006). For example, if the association between discounting and IGT performance is largely explained by differences in win/loss frequency sensitivity, we might expect weak associations when using standard IGT scoring procedures because the frequencies of wins and losses on the task are largely balanced across the good and bad decks.

## Reviewer G:

vii. P.7.: “More generally, computational model parameters provide more reliable and predictively valid measures of critical cognitive processes” ◊ this is overly general. Which exact computational models and parameters (!) are we talking about? The term is very broad and can encompass everything from a fancy regression to sophisticated Bayesian inference accounts with multiple levels of uncertainty, which surely differ in their reliability and predictive validity? Also perhaps depending on the experimental task they’re applied to?

## Author’s response

We agree this statement could be more precise. Through revising the text, this sentence was removed. However, the sentiment regarding the fact that computational parameters, particularly reinforcement learning parameters, are highly task-dependent is an important one that we discuss as part of the limitations as this would certainly apply to the present study. See pp. 34-35.

## Reviewer G:

viii. P.8.: “Thus, these studies show the utility of joint modeling for not only assessing test-retest reliability, but also for assessing convergence (or divergence) across psychological constructs.” I buy the argument but as mentioned above I’m missing some more specific arguments as to how the authors think reward valuation and learning from rewards and punishments might be related and why, and what might be aspects that differentiate between them. What are the authors’ hypotheses?

## Author’s response

We initially predicted that steep discounting would be associated with worse IGT performance, broadly, but that steep discounting would also be associated with elevated learning rates. These were, admittedly, not particularly strong predictions, so they were not included in the previous draft. We now mention these hypotheses in the text but emphasize that the analyses were not strongly driven by these hypotheses. Instead, this study was guided by a general desire to understand associations between delay discounting and specific patterns of behavior on the IGT that could contextualize previous findings illustrating associations between discounting and summary measures of IGT performance.

pp. 8-9: Broadly speaking, we hypothesized that people who showed steep discounting would perform worse on the task; however, computationally, we considered the possibility that steep discounting would be specifically associated with elevated reward and punishment learning rates. Specifically, if we assume steep discounting indexes sensitivity to immediate consequences that would be reflected at the trial-level on the IGT, we would expect steep discounting to be associated with elevated reward and punishment learning rates as these parameters characterize how much people weight trial-level consequences in updating the value of a chosen option. However, an elevated punishment learning rate, in particular, is associated with better performance on the play-or-pass IGT (see supplement for simulation study illustrating this pattern). Therefore, if participants who show steep discounting perform worse on the task, as expected, this could be due to an insensitivity to trial-level losses resulting in higher losses over time. Ultimately, our goal was to employ the ORL to identify specific patterns of behavior on the IGT, characterizing learning on the task, that may be differentially associated with steep delay discounting.

## Reviewer G:

2B) Methods

i. P.8.: The authors state that data from the play-or-pass IGT have been presented elsewhere. Can they clarify whether this means that they are republishing data? If so, can they clarify where the data were published? Also, do the authors see any benefit in waiting for the longitudinal data to come in and publishing it together rather than bit by bit?

## Author’s response

IGT data from this study have been previously published. We now explicitly state this in the Introduction (see excerpt below). In regards to the longitudinal data, these are presently being processed as part of another publication in preparation. This is a large dataset that is generating multiple manuscripts, where the focus differs across the offspring and/or the parents. The IGT data has previously been published, examining associations between history of psychopathology and IGT performance at the initial timepoint using the ORL model. The DD data has not been previously published. We have programmatic plans of examining these data with cross-sectional associations and full longitudinal models. The challenge is that the fitting of a full model, including both tasks on up to five occasions is technically difficult. Thus, building and troubleshooting the implementation of those models takes much time. That said, we appreciate the concern and we do not have ambitions of using these data to examine 2, then 3, then 4, then 5 timepoint models. We fully agree that conducting those minor incremental models would be highly problematic.

p. 10: [W]e used an existing dataset in which participants completed an intertemporal choice task and the play-or-pass IGT. Data from the play-or-pass IGT have been previously published (Haynes et al., 2025).

## Reviewer G:

ii. P.9.: Can the authors clarify whether there were in- and exclusion criteria for the parents’ children only or whether there were specific in- and exclusion criteria for the parents as well? And please indicate whether and how the authors think these criteria affect their adult sample, can these be understood as “healthy adults”? Most importantly, can the authors clarify whether for some families, both mother and father were included in the study and whether this was accounted for in the analyses (as these are then no longer independent from one another).

## Author’s response

The only criteria for excluding parents was a lifetime history of bipolar spectrum disorder and psychosis spectrum disorder. We note this more directly in the main text:

p. 11: [P]arents with a lifetime history of bipolar spectrum disorder or psychosis spectrum disorder were not eligible for participation and led to removing the family from the study.

With respect to whether the sample can be understood as healthy adults, participants with a past and present history of DSM-5 diagnoses were included. Generally, present history of a diagnosis was rare except for anxiety (*n* = 65). To avoid redundant publication, we focus this manuscript on the associations between discounting and IGT performance, and we include a statement of transparency noting this:

p. 10: For transparency, it should be noted that the previous study examined history of psychopathology on IGT performance and found that people with a current and past history of anxiety (combined) tend to show elevated punishment learning on the task (see Haynes et al., 2025 for details). The focus of this study was to clarify if and how delay discounting may be implicated in computational measures of learning on the play-or-pass IGT.

Finally, in regard to the issue of non-independence, we ran correlations between parameters from participants who were part of mother-father dyads included in this study. These correlations were in the low-to-moderate range (all *r*-values < .4), but we did observe a significant correlation between mother and father log *k* values (*r* = .36, *p* = .002). Generally, collinearity among parameters is problematic with higher r-values: e.g., Dormann et al. (2013) suggest *r*-values > |.7| are cause for concern. Given that correlations between mother-father parameters were well below this value, we consider this to be little concern in the present study. However, to take a conservative approach, we conducted a follow-up analysis where we randomly removed one participant from each mother-father dyad in the sample and refit the joint model. Although we generally replicate the finding that steep discounting is associated with lower punishment learning rates, this finding is statistically weaker (the *γ_A_pun_1_* coefficient is lower). Visually, however, we believe the association between steep discounting and lower punishment learning rates is reliable (see Figure S10). We do not replicate the association with win/loss frequency sensitivities. In our previous submission, we urged caution in interpreting the association between discounting and win/loss sensitivity because this association is weak (*r* = -.21); thus, this finding supports our urge of caution. We present this analysis in the main text as part of the Sensitivity Analyses (p. 26), with details specified in the supplement (pp. 14-16).

## Reviewer G:

iii. P.9.: The authors mention self-report questionnaires, but they never seem to crop up. Given that they investigate DD and learning as hypothesized etiological factors for mental health issues, could some of these questionnaires be relevant here? What were they?

## Author’s response

We appreciate the reviewer’s interest in including the self-report questionnaires. Given the manner in which we have restructured the manuscript - that is, focusing on the associations between delay discounting and patterns of behavior on the IGT explaining prior associations - we exclude these because we believe including them would detract from the overarching goals of the manuscript.

## Reviewer G:

iv. P.10.: Can the authors state how many trials there were in the IGT?

## Author’s response

We now include the number of trials and explicitly state that each deck was presented an equal number of times.

p. 12: Participants completed 120 trials with each deck presented 30 times each.

## Reviewer G:

v. P.11.: Why do the authors show the parameters in centered rather than native space?

## Author’s response

We clarify that this was for concision and clarity.

p. 13: For concision and clarity, we present the centered parameterizations of the person level parameters below; however, the models were implemented using equivalent non-centered parameterizations to increase efficiency and model convergence (Stan Development Team, n.d.).

## Reviewer G:

vi. P.11.: I think the reasoning behind the choice of priors should go in the main manuscript.

## Author’s response

p. 19: We have included a section on the choice of priors in the main text.

## Reviewer G:

vii. P.11.: Please report correlations between simulated and observed data and show scatter plots.

## Author’s response

Thank you for pointing this out; they were inadvertently omitted from the initial submission. See p. 3 of the supplement.

## Reviewer G:

viii. P.15.: “To further contextualize the between-task associations, we calculated follow-up correlations between discounting parameters and ORL parameters, and between discounting parameters and behavioral measures of IGT performance.” Please comment on whether/to what extent that constitutes double dipping if the slope is already calculated as part of the model. Wouldn’t the (standardized) average slope be the same as the correlation?

## Author’s response

This is correct. We only provide these correlations as we find them easier to interpret than the slope coefficients. We have clarified in the text that the correlations carry the same information as the *γ_1_* parameters.

p. 21: These correlations are presented purely for ease of interpretation, but they otherwise provide the same information as the *γ_1_* parameters.

## Reviewer G:

ix. P.16.: Since the ORL is rather central in the paper, it would be good to have a more intuitive explanation as to what it does in addition to the maths in table 2. Can the authors also comment on whether they considered other models for the DD and IGT tasks and, if not, why not? E.g., are the response bias / win loss frequency sensitivity / differential LRs for wins/losses really necessary; is there a choice stochasticity/sensitivity term? If not, why not etc.

## Author’s response

This comment is similar to Reviewer 1’s comment above (point 1E); therefore, further details of our response can be found above. In brief, we fit two simpler RL models to the IGT data: one model with a single learning rate for both gains and losses and another model with a learning rate for gains and a learning rate for losses. The ORL model outperformed these simpler RL models; therefore, we present results from the ORL. For the delay discounting model, we considered alternative models. Ultimately, we used the single-parameter hyperbola because of its widespread use and because this was the model employed by Kvam et al. (2021) from whom we based our approach on. In addition, although not discussed in the text, there is concern regarding collinearity among parameters in hyperboloid discounting models with multiple parameters (e.g., Rzeszutek et al., 2022: Notes on Demand: Conceptual and Empirical Benefits of Applying Rachlin’s Discounting Equation to Demand Data).

The use of the single-parameter hyperbola is noted as part of the limitations on p. 33: We used a simple delay discounting model to characterize discounting, but alternative models could be used to measure other decision-making processes on intertemporal choice tasks. We chose the hyperbolic discounting model because of its common use (e.g., Fontes et al., 2025; Klein et al., 2022; Kohler et al., 2022) and because this was the model used by Kvam et al. (2021) from whom we based on our joint modeling approach on.

## Reviewer G:

2C) Results

i. P.16.: “Overall, steep delay discounting (i.e., a higher log k) was associated with lower reward and punishment learning rates (Arew & Apun), win/loss frequency sensitivities (βf), and response biases (βb).” - have the authors checked whether this association is at all related to model fit? As in, might participants with higher log k just behave more randomly on the IGT?

## Author’s response

This is a insightful point. We conducted a follow-up analysis to determine whether participants who showed steep discounting were more difficult to predict data for on the IGT. More details can be found in the supplement (pp. 10-11), but to briefly summarize, there is a significant correlation between steep discounting and lower predictive performance, *r_S_* = –.19, *p* = .001; however, the correlation is rather weak and the scatterplot illustrating this association is not particularly convincing. That notwithstanding, this does not negate the reviewer’s point that steep discounting could reflect participants being insensitive to the consequences on the IGT. Our finding that steep discounting was associated with smaller changes in playing on the bad decks from the beginning of the task to the end of the task supports this possibility. We note this possible interpretation in the main text on pp. 28-29: An alternative interpretation of our data could be that people who show steep discounting may be generally insensitive to feedback on the IGT and essentially behave randomly on the task. In line with this possibility, we found that people who show steep discounting also showed less change in playing on the bad decks between the first and last blocks of the IGT. Likewise, when we assess correlations between log k values and person-level predictive performance of the ORL, we find that people who show steep discounting are more difficult to predict data from in the IGT; however, this association is rather weak (see supplement for further details).

## Reviewer G:

2D) Discussion

i. P.19. My understanding is that RL parameters tend to generalize poorly across tasks (with the exception of the inverse temperature/ decision noise/ choice stochasticity/ choice sensitivity parameters). Can the authors comment on what this means with regard to inferences from this specific study employing a specific version of the IGT to learning in general as well as the PVS and NVS globally?

## Author’s response

Thank you for pointing this out. This is similar to comments above (points 1B, 1C, & 2A.vii.). We have added this point as part of our limitations such that generalizing beyond our study should be made carefully given the issues associated with poor reliability and generalizability of RL parameters. See pp 34-35.

## Reviewer G:

ii. P.19.: Vague: “Overall, our findings suggest that learning on the IGT and reward valuation on ITC tasks contribute to overlapping patterns of behavior, advancing our understanding of the RDoC PVS and NVS” - as mentioned above wrt the introduction, I’m missing a bit of theory here. How do they relate and how does that make sense conceptually?

iii. P.19.: “our findings may be helpful in understanding how dysfunctions in these processes may contribute to mental illness” - callback to my point about questionnaires from earlier. Also, can the authors specify in what way their findings will be helpful? Not trying to be facetious, it would just be good to understand how a more complete picture of the interrelationships between DD and learning may help understand dysfunctions underlying psychiatric symptoms.

## Author’s response

Points 2D) ii. and 2D) iii. are similar; therefore, we respond to them together. Overall, through restructuring the manuscript, we shifted the focus from the broad PVS and NVS components of the RDoC towards understanding associations between discounting and patterns of behavior on the IGT. We still note the relevance of the PVS and NVS as this contextualizes why our findings may be important. To that end, we provide additional discussion of how our findings may be contextualized in terms of understanding mental illness.

p. 28: This may be helpful in understanding maladaptive behavior in certain forms of psychopathology, namely substance abuse. For example, Bickel et al. (2012b) have suggested that people who do not value delayed consequences, measured with steep discounting, are biased towards the short-term value of harmful reinforcers while neglecting the long-term negative consequences of those reinforcers. Generally, the finding that worse IGT performance is associated with steep discounting supports this perspective; however, our data suggest this may be due to a specific insensitivity to the magnitude and frequency of losses. Thus, it is possible that populations of people who show steep discounting (e.g., populations engaging in substance abuse) may be insensitive to the losses occurring over time that ultimately lead to larger negative consequences experienced later (e.g., incarceration, loss of social reinforcers with excessive drug use; Ignaszewski, 2021).

## Reviewer G:

iv. P.20.: “Our data supports this perspective, such that people who showed steeper discounting were less sensitive to punishment on the IGT in terms of loss magnitude (lower Apun) and loss frequency (lower βf).” … but also lower sensitivity to win frequency. So perhaps generally lower sensitivity to feedback? / more randomness?

## Author’s response

This is similar to point 2C) i above. We note this possible interpretation in the main text on pp. 28-29: An alternative interpretation of our data could be that people who show steep discounting may be generally insensitive to feedback on the IGT and essentially behave randomly on the task. In line with this possibility, we found that people who show steep discounting also showed less change in playing on the bad decks between the first and last blocks of the IGT. Likewise, when we assess correlations between log k values and person-level predictive performance of the ORL, we find that people who show steep discounting are more difficult to predict data from in the IGT; however, this association is rather weak (see supplement for further details).

## Reviewer G:

v. P.20. Have the authors considered looking at delay discounting for losses as well as wins? Would this not more directly test their hypothesis that “populations of people who show steep discounting […] may be insensitive to the losses occurring over time that ultimately lead to larger negative consequences experienced later”?

## Author’s response

Thank you for pointing this out. We actually have ongoing research examining this exact topic and may be relevant to the point described above regarding whether discounting relates to randomness on the IGT. That is, we would expect that if discounting is related to insensitivity to losses, this should be reflected more strongly with discounting tasks that ask about losses.

P. 29: [W]e would expect steep discounting of losses would be more strongly associated with insensitivity to long-term losses on the IGT over and above steep discounting of gains. This is an important avenue for future research to explore, not just for more precisely identifying how discounting relates to learning (i.e., reduced punishment learning or insensitivity to feedback in general), but also in identifying alternative measures (i.e., measures of loss discounting) that provide better convergence with IGT performance.

## Reviewer G:

vi. P.21.: “dysfunctional reward and punishment learning are independently associated with psychopathology (e.g., substance abuse)” - is it true that they are clearly *independently* associated with psychopathology or just in separate studies? Either way this needs a reference.

## Author’s response

Through restructuring, this sentence was removed; however, we found that this may have been an issue with how we worded these associations in the Introduction and have clarified accordingly.

pp. 5-6: [P]oor performance on the IGT is associated with similar forms of psychopathology as delay discounting, including for example depression (Siqueira et al., 2018; Wang et al., 2024) and substance use (Barry & Petry, 2008; Petry et al., 1998; Verdejo-Garcia et al., 2007; see Bickel et al., 2019 for a review on delay discounting in psychopathology; see Mukherjee & Kable, 2014 for a review & meta-analysis on IGT performance in psychopathology).

## Reviewer G:

vii. In general, perhaps the authors might want to comment on whether delay discounting should always be considered irrational/impulsive. There might be environments where DD is in fact very rational - e.g., a high inflation environment, if the agent thinks they can invest the money more profitably than the game does, if the environment is unreliable (e.g., the money may not be paid out), if the agent’s horizon is diminished (due to terminal illness, living in a warzone etc.)… This might be good to clarify but also provide a handle on why some people might develop steep DD in the first place.

## Author’s response

This is a good point and the IGT may be an interesting task to illustrate this aspect of delay discounting. We added a brief discussion of this to the Discussion section.

p. 31: [I]f we arranged a gambling task in which lower punishment learning rates and win/loss frequency sensitivities were associated with better performance, would people who show steep discounting perform better or worse on the gambling task? At face value, our data predict the former; that is, steep discounting being associated with better IGT performance. Such a finding would connect to literature suggesting that what we label as “impulsive” or “suboptimal” is contextually dependent. For example, seemingly impulsive behavior like steep discounting is adaptive in environments where the likelihood of attaining a delayed outcome is lower (e.g., areas with high mortality rates & lower life expectancies; Daly & Wilson, 2005; Stevens & Stephens, 2010). Connecting to this literature could be helpful in understanding why steep discounting develops in the first place (i.e., as an adaptive response to the environment). Thus, identifying how delay discounting relates to (sub)optimal performance on tasks measuring reward and punishment learning, like the IGT, could help us identify the contexts that favor steep discounting.

# Reviewer report: Round 2, Author Response

**DOI:** 10.5334/cpsy.172.r6

Thank you for the opportunity to revise our manuscript. Below, we list reviewer comments (bolded) along with our responses (unbolded). We provide excerpts from the manuscript to illustrate some of the changes (blue font). In addition, upon inspecting the raw data, we found that one participant’s anonymous ID was incorrectly coded for the delay discounting data which inadvertently led to their removal in the data processing stage and thus also in the model-fitting stage. We reran all analyses with this participant’s data included. Results were qualitatively identical with this participant; we only updated the associated numerical results accordingly, the majority of which was identical.

**Reviewer 1:**

## Reviewer A:

The only remaining point is that I recommend that the authors present statistical analyses of the empirical play proportion data, in particular, whether the different decks differ in their play probabilities (Figure 1). In response to my earlier comment on this (comment #ii), the authors added “posterior p-values” for the posterior predictive checks. However, my point was purely about the empirical data, where, so far, the play proportions are not statistically compared between the decks.

## Author’s response

This was a misunderstanding on our part and thank you for clarifying. We have included statistics on the empirical play proportion data.

p. 13 (Data Analysis section): We conducted a preliminary analysis to determine whether participants were sensitive to the long-term outcomes associated with the decks on the play-or-pass Iowa Gambling Task (IGT). To do this, we tested whether play proportions varied across decks with a generalized linear mixed model (GLMM) fit using the lme4 package (Bates et al., 2015). We used a binomial link function to fit the GLMM to the number of plays for each deck (out of 30 total trials for each deck). We included a fixed effect of deck (categorical) and random intercepts for participants: *Plays ~ Deck* + (1|*Participant*). We evaluated the significance of the fixed effect of deck with a Wald test using the car package (Fox & Weisberg, 2019) with Bonferroni-corrected follow-up comparisons conducted using the emmeans package (Lenth & Piaskowski, 2026). Results of the GLMM are presented with posterior predictive checks (see below for details) to support the visual analysis of the data.

p. 22 (Results section): On the IGT, participants were sensitive to the contingencies associated with each deck, such that across trials, participants avoided the bad decks (i.e., passed on decks A & B) and approached the good decks (i.e., played on decks C & D). The GLMM on the number of plays across decks found a significant main effect of deck χ^2^(3) = 972.22, *p* < .001. Follow-up comparisons showed that participants played on the good decks (decks C & D) more often than on the bad decks (decks A & B), all *p*s < .001. Among the two good decks, participants chose deck D, with the most frequent wins, more often than deck C, *p* < .001. In addition, among the two bad decks, participants chose deck A, with the most frequent losses, less often than deck B, *p* < .001.

## Reviewer B:

2A) Methods

ii. The reward learning rate parameter does not recover well (*r*=.48) - not sure if I would call this adequate recovery. Can the authors comment on how/whether this limits their interpretations and whether they have made attempts at improving the model to recover better?

## Author’s response

This is a good point. We temper the language regarding adequate recovery in the Method.

p. 14: Finally, we performed parameter recovery diagnostics, displayed in detail in the supplement. In general, parameters showed adequate recovery; however, reward learning rates from the Outcome Representation Learning model showed the poorest recovery (*r* = .48). Implications of this suboptimal parameter recovery are discussed in the Discussion.

In the Discussion, we describe how suboptimal parameter recovery for the reward learning rates may impact our results. In addition, we discuss our previous attempts to improve recovery for reward learning rates. First, we note that variations of the model (e.g., removing win/loss frequency sensitivity or using a single learning rate) have resulted in worse model fit (see supplement of current study). In addition, Haynes et al. (2024) introduced the current structure of the play-or-pass ORL, in part, to address poor parameter recovery of the reward learning rates for the original ORL applied to this task. They found correlations between true and recovered reward learning rates of *r* = .19 & .35 when estimated from a joint model assessing test-retest reliability across two sessions. Haynes et al. simplified the original ORL by replacing the memory decay and perseverance parameters with a response bias parameter (*βb*) and found improved recovery of reward learning rates. Like the present study, recovery of reward learning rates was still less than ideal (*r*s of .56 & .59); however, suboptimal parameter recovery for reward learning rates may be partially due to the task structure. Participants generally play frequently at the beginning of the task, and after encountering the losses on the bad decks, begin playing less on those decks. Thus, updating in response to gains, captured by reward learning rates may be difficult to estimate from play-behavior on this version of the IGT. Because of this, we recommend that researchers specifically interested in reward learning rates as a measure of individual differences against using the play-or-pass IGT. We expand on this further in the Discussion. Below we show an excerpt with bold underlining to highlight the main edits.

pp. 30-31: In addition to the findings noted above, our results suggest that delay discounting is not associated with response bias and reward learning rates on the IGT. Although further research may be needed to provide decisive evidence regarding the association between discounting and response bias (cf. the BF_10_ = 1.17), the lack of association between these parameters may not be surprising given that response bias characterizes a general tendency to play which may not have a strong theoretical interpretation (although see Haynes et al., 2025 for possible interpretations). The lack of association between delay discounting and reward learning rates, however, may be surprising because the intertemporal choice task involved choices about monetary rewards which, at face value, would be expected to relate to reward learning. **This finding may be due to reward learning rates being difficult to estimate from the ORL model, reflected by the suboptimal recovery of these learning rates (*r* = .48, see supplement). Although adjusting the structure of the play-or-pass ORL could improve estimation of reward learning rates, our lab has generally found that alternative formulations of the ORL perform worse (see supplement & Haynes et al., 2024). Instead, reward learning rates may be specifically difficult to estimate from this version of the IGT because of the typical patterns of behavior we observe on the task.** Participants typically play frequently at the beginning of the task, likely to gain information about the decks (see also Haynes et al., 2025). Across trials, participants adjust their behavior in response to the losses from the decks which would primarily be reflected by the punishment learning rates. Thus, we advise researchers specifically interested in how *reward valuation*, measured with delay discounting, and *reward learning*, measured with IGT-like decision-making tasks, against using the play-or-pass IGT. Alternative tasks, like the traditional IGT may be better suited for studying these associations, although further research would be necessary to confirm this possibility.

## Reviewer B:

2B) Results

i. From original response: We conducted a follow-up analysis to determine whether participants who showed steep discounting were more difficult to predict data for on the IGT. We found a significant correlation between steep discounting and lower predictive performance, *r_S_* = –.19, *p* = .001.

Reviewer: If the authors often come across correlations > .19 in their studies I congratulate and envy them, but my impression is that in psychology more broadly, *r*=.19 can hardly be called “rather weak”. If the authors insist on that wording, I would urge them to mention the exact number as well (as in “[…] is rather weak (*r*=.19)”). If space at all permits, I would also suggest the authors move this analysis to the sensitivity analyses in the main text.

## Author’s response

We removed “rather” from the text and simply state that the correlation was weak but significant. In addition, we moved the text from the supplement to the Sensitivity Analysis results section of the main text along with the figure (now Figure 6).

p. 27: Finally, a helpful reviewer pointed out that participants with steep discounting may be insensitive to feedback more broadly speaking, instead of insensitive to losses as our results suggest. Our finding that participants who demonstrated steep discounting showed little change in playing on the bad decks across the task supports this possibility. Another implication of this suggestion is that participants who show steep discounting may also be more difficult for the ORL model to characterize. To explore this, we assessed whether log k values were associated with how well the joint model predicted individual participants’ data from the play-or-pass IGT. For this, we calculated the proportion of predicted choices matching the observed choices on the IGT (labeled “proportion matched”), using the 16000 samples from the posterior predictive checks. Next, we correlated proportion matched with log k values. As illustrated in Figure 6, the association between proportion matched and log k values was weak; however, the correlation was statistically significant, *r_S_* = –.19, *p* = .001, suggesting that participants who showed steep discounting tended to be more difficult to predict data for on the IGT.

We also clarified this point in the Discussion section as well.

p. 29: An alternative interpretation of our data could be that people who show steep discounting may be generally insensitive to feedback on the IGT and essentially behave randomly on the task. In line with this possibility, we found that people who show steep discounting have lower win/loss frequency sensitivities, punishment learning rates, and although not statistically supported, lower reward learning rates (see Figure 2). Steep discounting was also associated with lower predictive performance on the IGT (*r* = -.19 between proportion matched & log *k*s) as well as less change in playing on the bad decks between the first and last blocks of the IGT (i.e., the difference score correlations).
